# Abundance, classification and genetic potential of *Thaumarchaeota* in metagenomes of European agricultural soils: a meta-analysis

**DOI:** 10.1186/s40793-023-00479-9

**Published:** 2023-03-30

**Authors:** Johanna Nelkner, Liren Huang, Timo W. Lin, Alexander Schulz, Benedikt Osterholz, Christian Henke, Jochen Blom, Alfred Pühler, Alexander Sczyrba, Andreas Schlüter

**Affiliations:** 1 Genome Research of Industrial Microorganisms, CeBiTec - Center for Biotechnology, Bielefeld University, Bielefeld, Germany; 2 Computational Metagenomics Group, CeBiTec - Center for Biotechnology, Bielefeld University, Bielefeld, Germany; 3 Machine Learning Group, CITEC - Cognitive Interaction Technology, Bielefeld University, Bielefeld, Germany; 4 Bioinformatics and Systems Biology, Justus-Liebig-University, Gießen, Germany; 5 Nucleic Acids Core Facility, Faculty of Biology, Johannes Gutenberg University Mainz, Germany Mainz,

**Keywords:** European soil, Agricultural microbiome, Open metagenome data analysis, Metagenomically assembled genomes, Soil health, *Thaumarchaeota*, Soil microbial diversity

## Abstract

**Background:**

For a sustainable production of food, research on agricultural soil microbial communities is inevitable. Due to its immense complexity, soil is still some kind of black box. Soil study designs for identifying microbiome members of relevance have various scopes and focus on particular environmental factors. To identify common features of soil microbiomes, data from multiple studies should be compiled and processed. Taxonomic compositions and functional capabilities of microbial communities associated with soils and plants have been identified and characterized in the past few decades. From a fertile Loess–Chernozem-type soil located in Germany, metagenomically assembled genomes (MAGs) classified as members of the phylum *Thaumarchaeota/Thermoproteota* were obtained. These possibly represent keystone agricultural soil community members encoding functions of relevance for soil fertility and plant health. Their importance for the analyzed microbiomes is corroborated by the fact that they were predicted to contribute to the cycling of nitrogen, feature the genetic potential to fix carbon dioxide and possess genes with predicted functions in plant-growth-promotion (PGP). To expand the knowledge on soil community members belonging to the phylum *Thaumarchaeota*, we conducted a meta-analysis integrating primary studies on European agricultural soil microbiomes.

**Results:**

Taxonomic classification of the selected soil metagenomes revealed the shared agricultural soil core microbiome of European soils from 19 locations. Metadata reporting was heterogeneous between the different studies. According to the available metadata, we separated the data into 68 treatments. The phylum *Thaumarchaeota* is part of the core microbiome and represents a major constituent of the archaeal subcommunities in all European agricultural soils. At a higher taxonomic resolution, 2074 genera constituted the core microbiome. We observed that viral genera strongly contribute to variation in taxonomic profiles. By binning of metagenomically assembled contigs, *Thaumarchaeota* MAGs could be recovered from several European soil metagenomes. Notably, many of them were classified as members of the family *Nitrososphaeraceae*, highlighting the importance of this family for agricultural soils. The specific Loess-Chernozem *Thaumarchaeota* MAGs were most abundant in their original soil, but also seem to be of importance in other agricultural soil microbial communities. Metabolic reconstruction of Switzerland_1_MAG_2 revealed its genetic potential *i.a.* regarding carbon dioxide (CO$_2$) fixation, ammonia oxidation, exopolysaccharide production and a beneficial effect on plant growth. Similar genetic features were also present in other reconstructed MAGs. Three *Nitrososphaeraceae* MAGs are all most likely members of a so far unknown genus.

**Conclusions:**

On a broad view, European agricultural soil microbiomes are similarly structured. Differences in community structure were observable, although analysis was complicated by heterogeneity in metadata recording. Our study highlights the need for standardized metadata reporting and the benefits of networking open data. Future soil sequencing studies should also consider high sequencing depths in order to enable reconstruction of genome bins. Intriguingly, the family *Nitrososphaeraceae* commonly seems to be of importance in agricultural microbiomes.

**Supplementary Information:**

The online version contains supplementary material available at 10.1186/s40793-023-00479-9.

## Background

According to the Eurostats database (ISSN 2443-8219), 39% of the total land area of the EU is used for agricultural production [1]. Agricultural soils host a huge biodiversity, have a central role in nutrient cycling and play a key role in climate change mitigation. The European Soil Data Centre (ESDAC, https://esdac.jrc.ec.europa.eu/, European Commission, Joint Research Centre) sees a mid-term goal in improving soil structure to enhance habitat quality for soil biota and crops, to reduce high-density subsoils and to avert the loss of particulate organic matter. Since anthropogenic processes have severely perturbed the natural nitrogen and carbon cycle on earth, and a balance between soil productivity and environmental protection has to be achieved, microbial soil consortia members involved in the transformation of compounds have been subject to research in recent years [2, 3]. Likewise, identification of best management practices for arable soils is subject to numerous studies in recent years. Soil management strategies include for example fertilization, crop rotation schemes and tillage [4–8]. The importance of stable soil aggregates for enhanced crop growth and prevention of soil erosion is centuries-old knowledge. Long-term studies provided valuable insights and have shown that tillage methods, which are often used intensively in order to loosen the soil in standard agriculture, have a disrupting impact on soil structure [5, 9–16]. Furthermore, the connection of stable soil aggregates to the functional potential regarding production of agglutinating exopolysaccharides and lipopolysaccharides of the soil microbial community has been demonstrated [7].

Chernozem soils (sometimes referred to as Tschernosem or black soil) are considered as highly fertile and agriculturally productive [6, 17, 18]. The archaeal phylum *Thaumarchaeota* (*Thermoproteota* according to the GTDB taxonomy [19]) was shown to dominate the archaeal communities in studied black soils [4, 18]. Genomes of representatives belonging to the order *Nitrososphaerales*, a subordinated order of the phylum *Thaumarchaeota*, are characterized, among others, by presence of several genes encoding enzymes involved in the synthesis of different extracellular polymeric substances (EPS) [20]. This enhancement in EPS-producing potential was interpreted to reflect their ability to form biofilms. This is seen as a very successful ecological adaptation, as biofilm structures not only offer protection against environmental stress and nutrient limitation, but can also serve as a matrix for direct nutrient or electron exchange that facilitate biogeochemical cycling [20]. The phylum *Thaumarchaeota* comprises members known for their role in soil ammonia oxidation and thus, converting ammonia to nitrite and further to nitric oxide. Ammonia oxidation represents the first and rate-limiting step in the nitrification process, thus contributing to the cycling of nitrogen. Members of the *Thaumarchaeota* are also able to fix carbon dioxide. These properties enable their autotrophic growth in soil [21].

In a previous study analyzing the loess chernozem-type soil of the ’Magdeburger Börde’ (Saxony-Anhalt, Germany), we found that members of the archaeal phylum *Thaumarchaeota* are abundant; the subordinated genus *Nitrososphaera* was amongst the top five most abundant genera [4]. Corresponding metagenomically assembled genomes (MAGs) were predicted to possess intact *amoA* genes, encoding a subunit of the ammonia monooxygenase catalyzing ammonia oxidation. Presence of *amoA* genes in their reconstructed genomes suggests the capability to oxidize ammonia. Moreover, the predicted potential to produce phytohormone precursors hints at a plant-growth promotion (PGP) capability mediated by these MAGs. The soil in the German area ’Magdeburger Börde’ is known for its high fertility [6]. Therefore, we hypothesized that the soil community composition contributes to corresponding characteristics.

We were interested in the question, whether *Thaumarchaeota* members are also abundant in agricultural soils of other European locations and whether they are part of the core microbiome in European agricultural soils.

To address these biological questions, we conducted a meta-analysis by considering 16 relevant primary studies reporting on microbial communities of agriculturally used soils to estimate European soil effectors and effect sizes contributing to shaping of the microbial community composition. We aimed to assess ecological coherence of members of the phylum *Thaumarchaeota* in agricultural soil communities across Europe by analyzing abundance profiles derived from single-read classification of publicly available whole metagenome sequencing data. We analyzed abundance data of microbial communities on the taxonomic levels of phylum, family and genus in order to measure effects on low, medium and high resolution. Our scope was to find general similarities, but also differences in taxonomic composition, local peculiarities and specific differences in abundances of *Thaumarchaeota* members. To follow the question, whether *Thaumarchaeota* MAGs can also be reconstructed from European soil metagenomes, we applied an assembly and binning procedure to single read metagenomic sequencing data and mined the retrieved genomes for encoded soil beneficial functions.

## Material and methods

### Selection of metagenomic datasets representing agricultural soil microbiomes

**Fig. 1:**
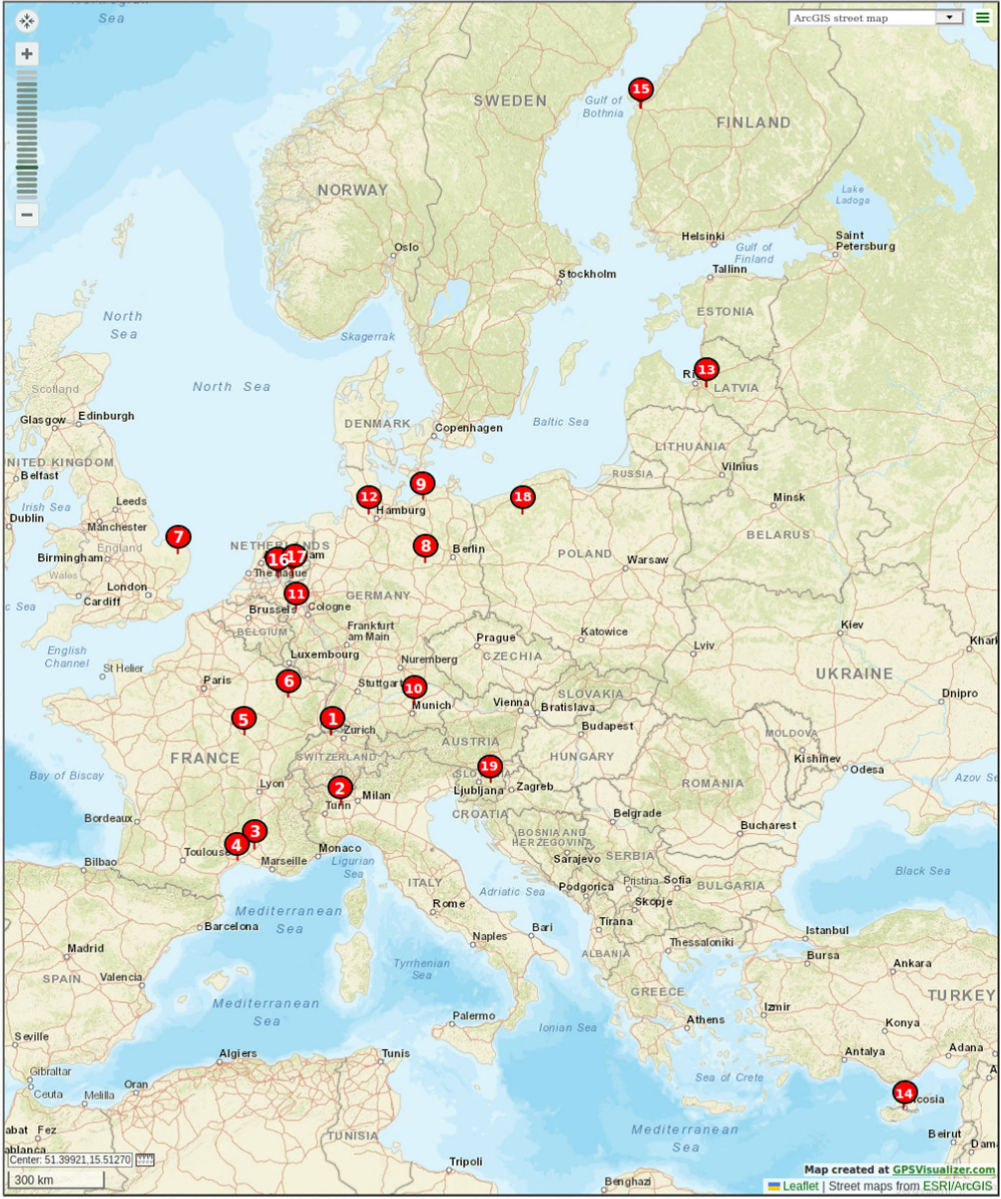
Geographic location of the origin of agricultural soil samples from the selected primary studies. The numbers refer to the location ID given in Table 1. Most soil samples are from locations in Central Europe. Soil from the ”Frick trial“ in Switzerland (location ID 1) was analysed in two independent studies. The location data was plotted using GPS Visualizer (https://www.gpsvisualizer.com/)

All SRA data (1.861.430 datasets, 30.09.2020) was copied to the CeBiTec / de.NBI compute cluster and searched using the in-house search engine ‘SRA metadata search’ by Christian Henke. All EU countries (Austria, Belgium, Bulgaria, Croatia, Republic of Cyprus, Czech Republic, Denmark, Estonia, Finland, France, Germany, Greece, Hungary, Ireland, Italy, Latvia, Lithuania, Luxembourg, Malta, Netherlands, Poland, Portugal, Romania, Slovakia, Slovenia, Spain and Sweden) were searched individually. The filter keywords were ‘$*$country soil metagenome illumina WGS’ (WGS $=$ whole (meta)genome shotgun sequencing). This search yielded 17 studies, which were further manually inspected for suitability. Only datasets with agricultural context, background or relevance and available corresponding peer-reviewed publications were selected. In total, 16 primary studies fulfilled the minimum standards. These 16 primary studies covered 20 soil origin locations, with the Frick trial in Switzerland being scope in two seperate primary studies [5, 22], therefore 19 different locations. We introduced the location tag (Table 1, first column) and plotted the locations of soil origins (Fig. 1) using GPS Visualizer (https://www.gpsvisualizer.com/). A detailed description of the used datasets and scopes of the primary studies is provided in the Additional file 1. The following SRA projects were included and downloaded from the European Nucleotide Archive (ENA) at EMBL-EBI: PRJNA387672, PRJNA393632, PRJNA378475, PRJNA550482, PRJEB12917, PRJNA390514, PRJEB31111,PRJNA385596, PRJNA557612, PRJNA532820, PRJEB15448, PRJEB35612, PRJNA518246-PRJNA518254, PRJNA488251, PRJNA435676, PRJNA555481.

**Table 1:** Selected studies divided into 68 treatments of soil microbiomes with agricultural context and availability of metadata

Location	Name	Primary	Crop	Compartment	Collection	Soil type	pH	Soil
ID		study			depth	simple		texture
1_1	Switzerland_CA	[5]	Green manure	Bulk soil	10 cm	Cambisol	7.1	Clayey
1_2	Switzerland_CB	[5]	Green manure	Bulk soil	20 cm	Cambisol	7.1	Clayey
1_3	Switzerland_CC	[5]	Green manure	Bulk soil	50 cm	Cambisol	7.1	Clayey
1_4	Switzerland_RA	[5]	Green manure	Bulk soil	10 cm	Cambisol	7.1	Clayey
1_5	Switzerland_RB	[5]	Green manure	Bulk soil	20 cm	Cambisol	7.1	Clayey
1_6	Switzerland_RC	[5]	Green manure	Bulk soil	50 cm	Cambisol	7.1	Clayey
2_1	Italy_BS_G	[56]	Rice	Bulk soil	10 cm	Cambisol*	6*	Sandy loam*
2_2	Italy_BS_noG	[56]	Rice	Bulk soil	10 cm	Cambisol*	6*	Sandy loam*
2_3	Italy_FFS	[56]	Rice	Bulk soil	NA	Cambisol*	6*	Sandy loam*
2_4	Italy_RS_G	[56]	Rice	Root-influenced	3 cm	Cambisol*	6*	Sandy loam*
2_5	Italy_RS_noG	[56]	Rice	Root-influenced	3 cm	Cambisol*	6*	Sandy loam*
3_1	France_1_IC	[4]	Asparagus	Root-influenced	NA	NA	NA	Sandy and Silty
3_2	France_1_FC	[4]	Asparagus	Root-influenced	NA	NA	NA	Sandy and Silty
3_3	France_1_ER	[4]	Asparagus	Root-influenced	NA	NA	NA	Sandy and Silty
3_4	France_1_ER_C	[4]	Asparagus	Root-influenced	NA	NA	NA	Sandy and Silty
4_1	France_2_MONT	[4]	Wheat*	Bulk soil*	NA	NA	8.6	Clay loam
5_1	France_2_EPO	[4]	Wheat*	Bulk soil*	NA	NA	5.6	Silty clay
6_1	France_3_BS	[4]	Bulk soil	Bulk soil	NA	NA	7.25*	Sandy loam*
6_2	France_3_RS	[4]	Green manure	Root-influenced	NA	NA	7.25*	Sandy loam*
7_1	UK_T0	[4]	Green manure*	Bulk soil	NA	NA	6.7	Sandy*
7_2	UK_T7	[4]	Bulk soil*	Bulk soil	NA	NA	6.7	Sandy*
7_3	UK_T14	[4]	Bulk soil*	Bulk soil	NA	NA	6.7	Sandy*
8_1	Germany_1_BS-P-Int	[4]	Wheat	Bulk soil	25 cm	Chernozem	7.2	Silty loam
8_2	Germany_1_BS-P-Ext	[4]	Wheat	Bulk soil	25 cm	Chernozem	7.2	Silty loam
8_3	Germany_1_BS-CT-Int	[4]	Wheat	Bulk soil	25 cm	Chernozem	7.2	Silty loam
8_4	Germany_1_BS-CT-Ext	[4]	Wheat	Bulk soil	25 cm	Chernozem	7.2	Silty loam
8_5	Germany_1_RS-P-Int	[4]	Lettuce	Root-influenced	25 cm	Chernozem	7.2	Silty loam
8_6	Germany_1_RS-P-Ext	[4]	Lettuce	Root-influenced	25 cm	Chernozem	7.2	Silty loam
8_7	Germany_1_RS-CT-Int	[4]	Lettuce	Root-influenced	25 cm	Chernozem	7.2	Silty loam
8_8	Germany_1_RS-CT-Ext	[4]	Lettuce	Root-influenced	25 cm	Chernozem	7.2	Silty loam
9_1	Germany_2_HRO_C	[7]	Maize*	Bulk soil*	NA	Cambisol	6.3	Loamy sand
9_2	Germany_2_HRO	[7]	Maize*	Bulk soil*	NA	Cambisol	6.3	Loamy sand
10_1	Germany_2_FR_C	[7]	Maize*	Bulk soil*	NA	Cambisol	6.35	Silty loam
10_2	Germany_2_FR	[7]	Maize*	Bulk soil*	NA	Cambisol	6.35	Silty loam
11_1	Germany_3_RA_0	[7]	Bulk soil	Bulk soil	5 cm	Luvisol	7.5	Silty clay loam
11_2	Germany_3_RA_1	[7]	Alfalfa	Bulk soil	5 cm	Luvisol	7.5	Silty clay loam
11_3	Germany_3_RA_3	[7]	Alfalfa	Bulk soil	5 cm	Luvisol	7.5	Silty clay loam
11_4	Germany_3_RA_6	[7]	Wheat	Bulk soil	5 cm	Luvisol	7.5	Silty clay loam
11_5	Germany_3_RA_12	[7]	Maize	Bulk soil	5 cm	Luvisol	7.5	Silty clay loam
11_6	Germany_3_RA_24	[7]	Wheat	Bulk soil	5 cm	Luvisol	7.5	Silty clay loam
12_1	Germany_4_ARD_BS	[37]	Bulk soil	Bulk soil	20 cm	Luvisol	5.7	Loamy sand
12_2	Germany_4_ARD_RS	[37]	Apple plants	Root-influenced	20 cm	Luvisol	5.7	Loamy sand
12_3	Germany_4_CO_BS	[37]	Bulk soil	Bulk soil	20 cm	Luvisol	5.7	Loamy sand
12_4	Germany_4_CO_RS	[37]	Apple plants	Root-influenced	20 cm	Luvisol	5.7	Loamy sand
13_1	Latvia_PPS	[37]	Lettuce	Root-influenced	NA	Peat soil	6.23	Peat soil
13_2	Latvia_PPS_Chitin	[37]	Lettuce	Root-influenced	NA	Peat soil	6.03	Peat soil
14_1	Cyprus_BS_SC	[37]	Bulk soil	Bulk soil	NA	Sandy clay loam	8.6	Sandy clay loam
14_2	Cyprus_BS_S20	[37]	Bulk soil	Bulk soil	NA	Sandy clay loam	8.6	Sandy clay loam
14_3	Cyprus_BS_S100	[37]	Bulk soil	Bulk soil	NA	Sandy clay loam	8.6	Sandy clay loam
14_4	Cyprus_RS_EC	[37]	Lettuce	Root-influenced	NA	Sandy clay loam	8.6	Sandy clay loam
14_5	Cyprus_RS_E20	[37]	Lettuce	Root-influenced	NA	Sandy clay loam	8.6	Sandy clay loam
14_6	Cyprus_RS_E100	[37]	Lettuce	Root-influenced	NA	Sandy clay loam	8.6	Sandy clay loam
15_1	Finland_OX	[37]	Bulk soil	Bulk soil	75?cm	Holocene	3.7	Clayey
16_1	Netherlands_1_RS_At	[22]	*A. thaliana*	Root-influenced	10 cm	Loamy sand soil	NA	Loamy sand
16_2	Netherlands_1_RS_Zm	[22]	Maize	Root-influenced	10 cm	Loamy sand soil	NA	Loamy sand
16_3	Netherlands_1_RS_Ta	[22]	Wheat	Root-influenced	10 cm	Loamy sand soil	NA	Loamy sand
16_4	Netherlands_1_BS	[22]	Bulk soil	Bulk soil	10 cm	Loamy sand soil	NA	Loamy sand
17_1	Netherlands_2_BS	[22]	Bulk soil	Bulk soil	NA	Podzol	5.4	Sandy
17_2	Netherlands_2_RS_AtCm3	[22]	*A. thaliana*	Root-influenced	NA	Podzol	5.4	Sandy
17_3	Netherlands_2_RS_AtCp3	[22]	*A. thaliana*	Root-influenced	NA	Podzol	5.4	Sandy
17_4	Netherlands_2_RS_AtFm3	[22]	*A. thaliana*	Root-influenced	NA	Podzol	5.4	Sandy
17_5	Netherlands_2_RS_AtMm3	[22]	*A. thaliana*	Root-influenced	NA	Podzol	5.4	Sandy
1_7	Switzerland_2_ConvTill	[22]	Green manure	Bulk soil	10 cm	Cambisol	7.33	Clayey
1_8	Switzerland_2_RedTill	[22]	Green manure	Bulk soil	10 cm	Cambisol	7.08	Clayey
18_1	Poland_ConvTill	[22]	Lupine*	Bulk soil	10 cm	Arenosol	6.3	Sandy
18_2	Poland_RedTill	[22]	Lupine*	Bulk soil	10 cm	Arenosol	6.2	Sandy
19_1	Slovenia_2_ConvTill	[22]	Green manure	Bulk soil	10 cm	Cambisol	6.43	Loamy
19_2	Slovenia_2_RedTill	[22]	Green manure	Bulk soil	10 cm	Cambisol	6.95	Loamy

* Metadata that were derived with resources other than the original primary study, e.g. personal communication or secondary study, are marked with an asterisk [*]

### Metadata compilation

Crop categories were built to be as broad as possible, for example, if ryegrass, green manure ley or green manure mixtures were named as crops, we aggregated them to the category ‘green manure’. Thereby, we focused on actual crops and did not consider crop rotations. For the assignment of the compartment we combined root-influenced and true rhizosphere soil samples to the category root-influenced soil in order to have a broader category. For tillage annotations, if available, ploughed samples (depth $>=$ 15 cm) were determined ‘conventional tillage’, when the tillage depth was above 15 cm we annotated ‘reduced tillage’. If a range was given for metadata, e.g. soil pH, the average was taken. The soil texture triangle [23] was used to classify soil texture where the texture was not explicitly described but percentages of sand silt and clay were available. For the UK soil, the texture annotation was retrieved by searching the geographic coordinates in the Soilscape map (http://www.landis.org.uk/soilscapes/#).

The final metadata table is shown in Table 1, an extended version is available in the Additional file 2.

### Taxonomic classification and analyses of soil microbiomes

Taxonomic classification of single read metagenomic sequencing data was carried out using Kaiju [24]. The most comprehensive (within Kaiju’s options) reference sequence database, NCBI RefSeq [25], was used to present a sensitive taxonomic classification. A particular advantage of the Kaiju classifier is its higher sensitivity for genera that are underrepresented in the reference database [24]. For parameter settings, we set to allow a maximum of three mismatches in the alignment and a minimum match length of eleven nucleotides. To account for differences in sequencing depth and in order to ensure comparability between the datasets from different primary studies, we subsampled/rarefied the raw reads retrieved from SRA to one million reads per treatment prior to all single read based analyses using SparkHit’s subsampling function [26]. For samples with less than one million reads, the retrieved abundance values were normalized to one million reads.

### Assembly and binning of metagenome sequence data

The preprocessed reads were assembled using MEGAHIT (v1.2.9; preset: meta-large) [27]. Assembled contigs longer than 500 bases were further subjected to structural annotation using Prodigal (v2.6.3) [28]. The predicted coding sequences then were functionally annotated using DIAMOND (v0.9.36) [29] against the databases National Center for Biotechnology Information non-redundant protein sequences database (NCBI-nr) and KEGG (both with e-value cutoff 0.001), and using Hidden-Markov-Modell (HMM) search against Pfam (e-value cutoff 0.001). Reads were mapped back onto the assembly using BBMap (v38.86, Bushnell, http://jgi.doe.gov/data-and-tools/bbtools/). The assembled contigs were binned using MetaBat (v2.12.1) and, subsequently, metagenomically assembled genomes (MAGs) were classified according to the Genome Taxonomy Database [19] using GTDB-Tk (v1.3.0, https://github.com/Ecogenomics/GtdbTk). For exploration of calculated observations and in order to inspect functional annotations and binning results, assembled genes, contigs and MAGs were imported into the Elastic MetaGenome Browser (EMGB) platform [30]. EMGB is a fast web-based viewer for metagenomic analyses featuring various visualizations, filtering options and comparisons. The quality of the MAGs was determined by the metrics completeness and contamination as calculated by checkM (v1.0.12) [31]. We included *Thaumarchaeota* MAGs in the downstream analyses if their completeness was more than 50% and less than 10% contamination.

#### Estimation of MAG abundances via fragment recruitments of metagenome single reads

In order to generate abundance profiles of the MAGs in different soil metagenomic datasets, fragment recruitments were performed by application of the bioinformatics tool SparkHit [26]. Corresponding computations were scaled-up and parallelized by using the de.NBI Cloud compute cluster (https://www.denbi.de/cloud). As a fast and sensitive fragment recruitment tool, the so-called Sparkhit-recruiter was applied. This tool extends the FR-hit pipeline [32] and is implemented natively on top of the Apache Spark. The fragment recruitment option implements the q-Gram algorithm to allow more mismatches than a regular read mapping during the alignment, so that extra information is provided for the metagenomic analysis. SparkHit was applied on all soil metagenome FASTQ files that were downloaded from ENA. Randomly chosen 1 million reads of each FASTQ file were compared to all selected reference genomes. The alignment identity threshold was set to >97$\%$ to only identify closely related genomes. For *Thaumarchaeota* fragment recruitments, the genome database from NCBI was filtered for complete reference genomes, yielding 18 genomes.

#### Phylogenetic analyses and genome mining of metagenomically assembled genomes (MAGs)

The publicly available *Thaumarchaeota* complete reference genomes and the *de novo* constructed MAGs were added to a private project in the EDGAR 3.0 platform for comparative genomics [33]. The constructed phylogenetic tree was exported in Newick format and visualized within Evolview v3 [34]. Unique genes (singeltons) were calculated within EDGAR 3.0 by grouping the most complete MAGs of the new genus (Italy_MAG_67 and Italy_MAG_183) to a metacontig using core genome calculation, TA-21 assigned MAGs to a metacontig (pan genome) and *Nitrososphaera* MAGs and reference genomes (pan genome calculation), and calculating the singeltons for the new genus group. Within EDGAR 3.0, the annotated genes were searched for C-cycling, N-cycling and PGP genetic determinants. Identification of carbohydrate-active enzymes encoded in MAGs was done by applying the web server and DataBase for automated Carbohydrate-active enzyme Annotation dbCAN [35]. Metabolic pathways of MAGs were predicted as described previously by Nelkner et al. [4]. Briefly, MAG-encoded gene products were mapped to KEGG (Kyoto Encyclopedia of Genes and Genomes, https://www.genome.jp/kegg) pathway maps. The corresponding functionality is also implemented in the Elastic Metagenome-Browser platform EMGB [30]. Within EMBG, KEGG pathway maps were visualized for selected MAGs with encoded enzymes being highlighted in the pathway.

## Results and discussion

### Geographic location of soils and compilation of corresponding metadata

In total, 16 primary soil metagenome studies publicly available in the Sequence Read Archive (SRA) fulfilled the minimum standards which were defined to be required for this meta-study. All selected studies refer to soil microbiomes of agricultural relevance; corresponding metagenomes were sequenced applying the Illumina technology and publications are available (Table 1). A detailed description of the selected datasets, their grouping into soil treatments and scopes of the primary studies are provided in Additional file 1.

The geographic location of the studied soil origins is indicated in Fig. 1: Most soil samples were taken in Central Europe. Soil metadata was partially available for the following environmental parameters: geographic location, soil type, soil texture, soil composition ($\%$ sand, silt and clay), cultivated crop, compartment (bulk soil or root-influenced soil), tillage, fertilization, sampling depth, annual precipitation, soil pH and soil organic content. However, metadata reporting was inconsistent and heterogeneous between the different studies. For some metadata, like compartment, we were able to deduce an assignment, for others, for example pH, tillage or fertilization, we contacted the corresponding authors, but not in all cases those metadata were collected or available. In order to enhance comparability, we combined, where possible, metadata into higher categories. Unfortunately, in almost none of the studies, soil productivity, by means of agricultural productivity or biomass yields measured in dry matter weight, was reported. Soil productivity would have been a parameter that could have allowed predictions on soil health, since soil productivity can be seen as an indicator thereof and is of great relevance in the context of food production. The compiled metadata table (Table 1) was used as the basis for our meta analyses.

### Taxonomic diversity of selected European soil microbiomes

#### General taxonomic composition of the microbial soil communities

**Fig. 2:**
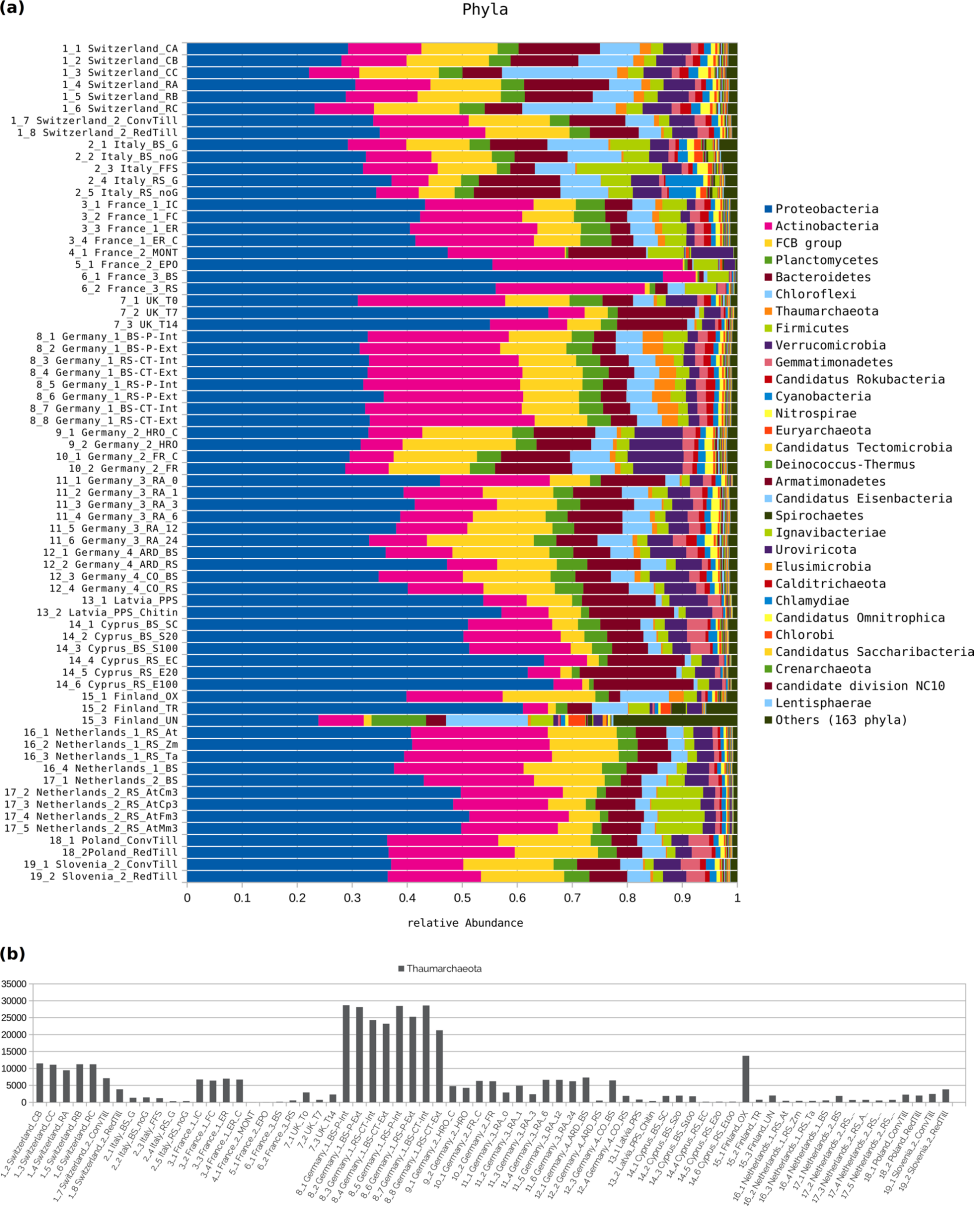
Phylum-level taxonomic profiles based on high-throughput metagenome single sequence-reads of the microbial soil communities divided into 68 treatments as specified in Table 1. **a** The top 30 phyla sorted by abundance in the Germany_1 study are colored; 163 other phyla with lower abundances are summed up (dark green bar on the right). **b** The bar plot shows the abundance of the phylum *Thaumarchaeota * (orange bar in the taxonomic profile above) in the European soils per treatment

It is generally known that healthy soils are characterized by high microbial diversity. In order to determine the diversity in the selected soil locations, the respective microbiomes were profiled taxonomically on the basis of the downloaded single metagenomic sequence reads. Taxonomic profiling was done for one million reads per treatment using the Kaiju classifier in its sensitive mode. Since we assume a contribution of *Thaumarchaeota* members to soil health and fertility, obtained taxonomic profiles were searched for taxa belonging to this phylum. The general compositions of the derived taxonomic profiles (Fig. 2a) are in accordance and comparable to those published for agricultural soil microbiomes [36]. Except for France_3 and Finland, the phylum level taxonomic profiles are similar. Bacterial phyla predominantly represented in the European soils include *Proteobacteria*, *Actinobacteria*, *FCB group*, *Planctomycetes*, *Bacteroidetes*, *Chloroflexi*, *Firmicutes*, *Verrucomicrobia*, and many more. *Thaumarchaeota*, *Euryarchaeota*, and *Crenarchaeota* represent the dominant archaeal phyla. Comparing all analyzed EU soil locations, the phylum *Thaumarchaeota* shows the highest abundance in the soil from the location ‘Bernburg’ (Germany_1), where it is the seventh most abundant phylum (Figs. 2a and 2b). *Thaumarchaeota* dominating the archeal subcommunity have been observed for Chernozem soils before [18]. Abundance of *Thaumarchaeota* seems to be higher in the upper soil layer, based on the Finnish study (Fig. 2b, Finland_OX). With higher depth, the availability of oxygen in the soil decreases and therefore might be suboptimal for the aerobic *Thaumarchaeota*. Further, the soil layers differ highly in soil pH. While in the Finland_OX sample, the authors reported a pH of 3.7, the pH in the Finland_TR and Finland_UN are at 4.7 and 8.1, respectively [37]. Therefore, both oxygen availability and pH might have an impact on *Thaumarchaota* abundance. For the dataset Germany_4, the *Thaumarchaeota* abundance shows differences between bulk soil and rhizosphere soil, with higher abundances in bulk soil samples. However, *Thaumarchaeota* members may represent very different species and therefore, it is important to also assess their abundance at lower taxonomic ranks [38].

#### The core microbiome of European agricultural soil microbial communities

Defining the core microbiome of all European soils can facilitate discrimination of the stable and permanent members of a microbiome from unique taxa that may be restricted to specific environmental conditions [39].

The core microbiome of all soils, defined by occurrence in all 68 distinguished samples consists of 153 phyla, 485 families and 2074 genera. In total, 193 different phyla were detected in all soils combined; in the median there are 189 phyla per treatment, with a maximum of 192 phyla (Switzerland_CA) and a minimum of 171 phyla per sample (France_2_MONT). The phylum *Thaumarchaeota* is part of the core microbiome and represents a major taxon of the archaeal subcommunities in the European agricultural soils.

**Fig. 3:**
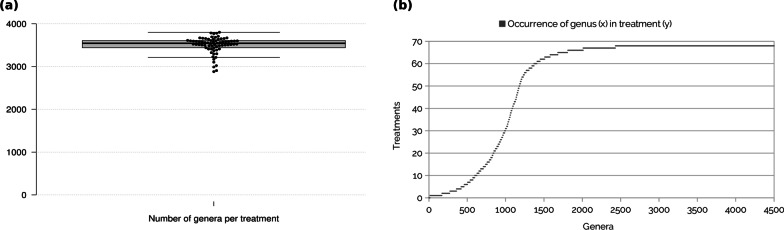
Statistics of diversity of the selected agricultural soil microbiomes. **a** Number of genera per soil treatment. The center line shows the median (3543 taxa per sample). The most diverse treatment counts 3802 genera (Germany_2_HRO_C), the least diverse treatment 2881 genera (Cyprus_RS_E100). Box limits indicate the 25th and 75th percentiles as determined by R software; whiskers extend 1.5 times the interquartile range from the 25th and 75th percentiles, data points are represented by dots; width of the boxes is proportional to the square root of the sample size; n $=$ 68 data points. **b** Prevalence of genera per treatment. For each of the 4508 genera on the x-axis a scatter is plotted representing the number of treatments out of the total 68 treatments it is prevalent. The data was sorted by prevalence. The Scatterplot shows an accumulation of data points at 65–68 treatments, meaning that a large proportion (46%) of the 4508 identified genera occurs in all 68 treatments and constitutes the core microbiome. For genera occurring in one to ten treatments, also an accumulation is visible. These are the genera that represent specialists, which are typical or specific for a treatment or group of treatments

In total, 4508 genera were detected. Figure 3a shows the distribution of the number of genera per sample. The median is at 3541 genera. The most diverse sample (Germany_2_HRO_C) counts 3802 genera. 2074 genera were present in all 68 samples (core microbiome) and 2925 genera in 65 or more samples, visible as a dense upper layer in the scatterplot shown in Fig. 3b. Interestingly, genera occurring in less than 55 samples are almost exclusively (84$\%$) viral genera. Recently, it has been shown that *Thaumarchaeota* virus populations carry thaumarchaeal ammonia monooxygenase genes (*amoC*) that were acquired via horizontal gene transfer from their host [40]. AmoC is a subunit of the ammonia monooxygenase responsible for ammonia oxidation from which *Thaumarchaeota* derive energy [41]. The observation, that the viral subcommunities are specific for certain soil habitats while prokaryotic communities are mostly ubiquitous, raises new research questions to address in order to unravel the enormous complexity of host-virus pairs and their ecological significance.

#### Distribution of *Thaumarchaeota* subtaxa

**Fig. 4:**
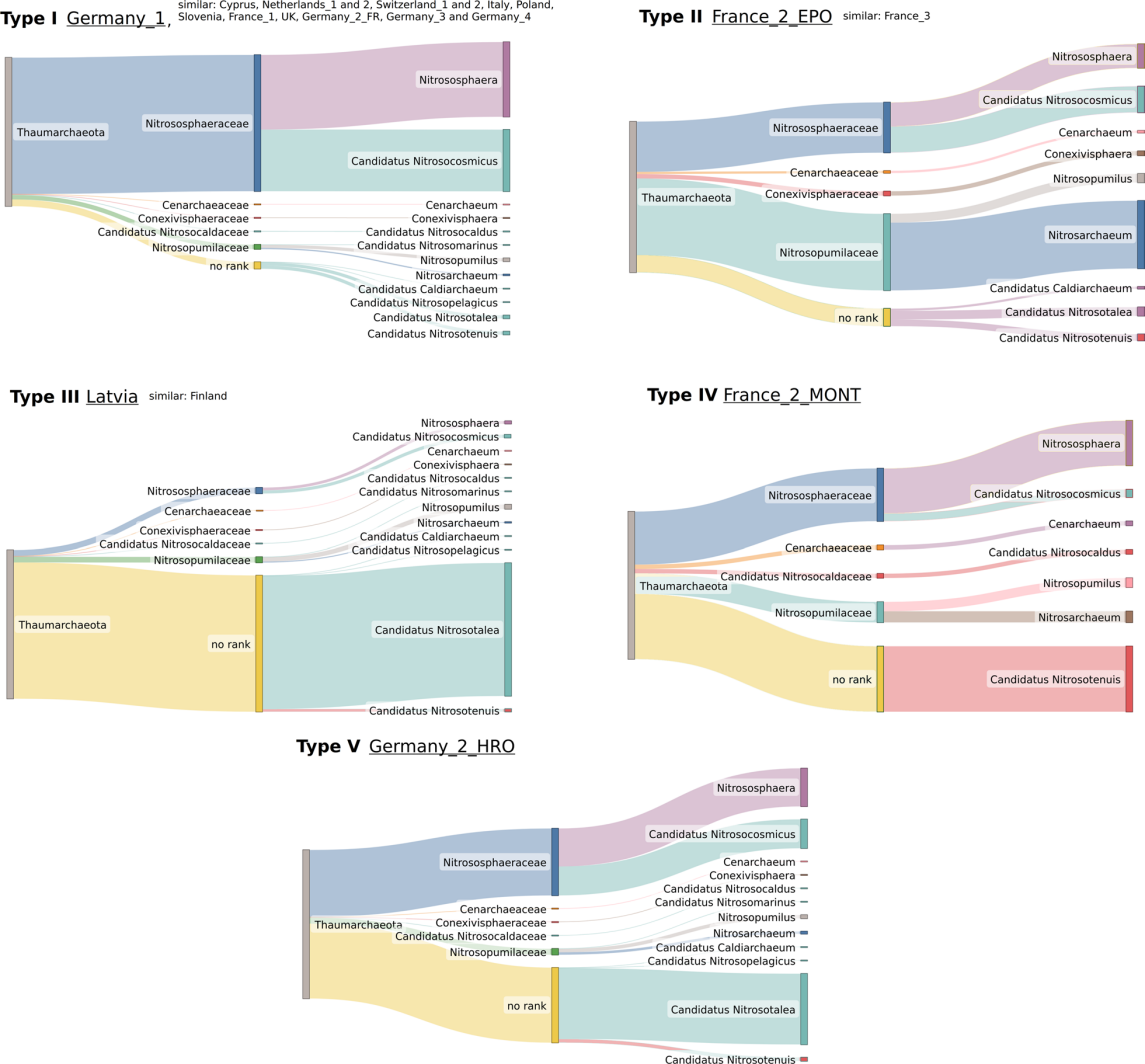
Distribution of taxa belonging to the phylum *Thaumarchaeota *per location shown for five representative distribution types. The Germany_1 distribution profile is representative for Cyprus, Netherlands_1, Netherlands_2, Switzerland_1, Switzerland_2, Italy, Poland, Slovenia, France_1, UK, Germany_2_FR, Germany_3 and Germany_4. Distribution of *Thaumarchaeota *subtaxa is similar in Latvia and Finland, further the distribution profile of France_3 resembles the profile of France_2_EPO. The profiles of France_2_MONT and Germany_2_HRO are rather unique. The similarity of distribution profiles was determined by visual inspection. In Additional file 3 all profiles are shown (treatments per location combined). On the left band, the Sankey diagrams show the phylum, which splits into families (middle) and further into genera (right). The widths of the bands are linearly proportional to the relative abundance within the soil locations, but the initial bands (phylum *Thaumarchaeota*) do not correspond to their relative abundance. The relative abundance of *Thaumarchaota* is shown in the bar plot in Fig. 2b. Sankey diagrams were created using SankeyMATIC (https://sankeymatic.com/)

Environmental effectors may affect only certain taxonomic groups. Gradually zooming into different levels of taxonomic assignments allows to observe substructures not visible on Phylum level, which can then be reflected in biogeochemical processes. The following families belonging to the phylum *Thaumarchaeota* were detected: *Nitrososphaeraceae* and *Nitrosopumilaceae* are prevalent in all 68 samples, *Cenarchaeaceae* in 66 samples, *Conexivisphaeraceae *and Candidatus *Nitrosocaldaceae* in 64 samples. Since the taxa distribution profiles are almost identical between treatments of the same location (data not shown), we analysed the distribution profiles per soil location. Further, since most distribution profiles had highly similar patterns (Additional file 3), we compiled them into types for clearer visualization. In most soil locations (13 of 19), the distribution of *Thaumarchaeota* subtaxa is similar and represented by pattern type I (Fig. 4). At genus level, the taxa *Nitrososphaera* and Candidatus *Nitrosocosmicus* dominate the representation of the *Thaumarchaeota* phylum in soils with subtaxa distribution profiles of type I. Some pronounced differences are apparent in the Latvia and Finland (type III), and Germany_2_HRO (type V) samples, where most of the thaumarchaeotal subcommunity is made up of the taxon Candidatus *Nitrosotalea*. As the available metadata of the soils from these locations are divergent, we were not able to deduce a hypothesis concerning occurrence of the latter taxon. In the Montpellier soil from the France_2 study (designated type IV), Candidatus *Nitrosotenuis* is the most abundant known *Thaumarchaeota* member. The genus *Nitrosarchaeum* is most abundant in the soil from Epoisses (France_2_EPO) and France_3 (type II). In this context too, the availability and heterogeneity of metadata complicate the formulation of a hypothesis.

### Reconstruction of metagenomically assembled genomes belonging to the phylum *Thaumarchaeota*

#### Assembly and binning results of the selected soil metagenome datasets

In order to access the most prominent microbial genomes, we pooled the single read metagenome sequencing data into groups based on their soil location. These groups were subjected to the EMGB assembly and binning pipeline. In total, we have successfully assembled 19 datasets. Table 2 shows the assembly and binning statistics. Cyprus and Germany_1 yielded the largest assemblies with 21 Gigabases (Gb) and 15 Gb, respectively.

**Table 2:** Assembly statistics of European agricultural soils metagenomic sequencing data

Assembly	No. of raw reads	Reads surviving	Assembly size	No. of	N50	reads mapping	No. of
		preprocessing	(bases)	contigs		back (median)	bins
Switzerland_1	19,655,846	15,324,319	394,292,388	523,285	706	22.34%	3
Italy	4,610,524,854	325,020,114	4,218,837,745	4,013,360	1,071	11.90%	274
France_1	43,885,434	39,598,532	218,973,513	285,653	2,300	10.63%	5
France_2_MONT	6,899,456	3,827,469	6,875,231	7,701	29,900	80.09%	0
France_2_EPO	37,607,912	21,299,224	108,210,514	86,089	38,100	85.84%	7
France_3	64,207,418	53,811,155	818,022,141	689,087	35,700	86.21%	93
UK	23,070,743	16,150,252	98,963,713	109,390	862	22.64%	10
Germany_1	252,545,422	222,340,015	15,095,628,983	16,987,507	870	67.82%	71
Germany_2_HRO	13,669,334	10,814,927	142,707,839	218,316	1,300	8.45%	0
Germany_2_FR	12,664,828	9,906,458	182,733,943	260,021	2,100	13.91%	1
Germany_3	22,903,380	16,803,398	218,286,828	341,269	1,100	11.69%	0
Germany_4	26,545,532	na	na	na	na	na	na
Latvia	225,414,876	26,801,474	1,342,086,652	1,300,992	1,033	23.80%	89
Cyprus	1,530,752,344	1,040,184,734	21,110,196,006	17,703,934	1,310	67.33%	1,508
Finland	1,887,593,101	1,340,782,681	13,822,566,779	9,774,981	1,791	75.19%	na
Netherlands_1	616,237,073	321,404,802	14,733,110,980	10,972,195	1,629	54.73%	106
Netherlands_2	549,290,648	138,320,059	470,252,336	411,467	39,500	73.29%	20
Switzerland_2	7,291,290	6,269,183	111,921,494	162,781	657	13.15%	0
Poland	8,722,704	7,665,665	85,452,227	134,128	612	8.41%	0
Slovenia_2	8,755,087	7,603,582	78,808,821	124,014	610	7.61%	0

**Fig. 5:**
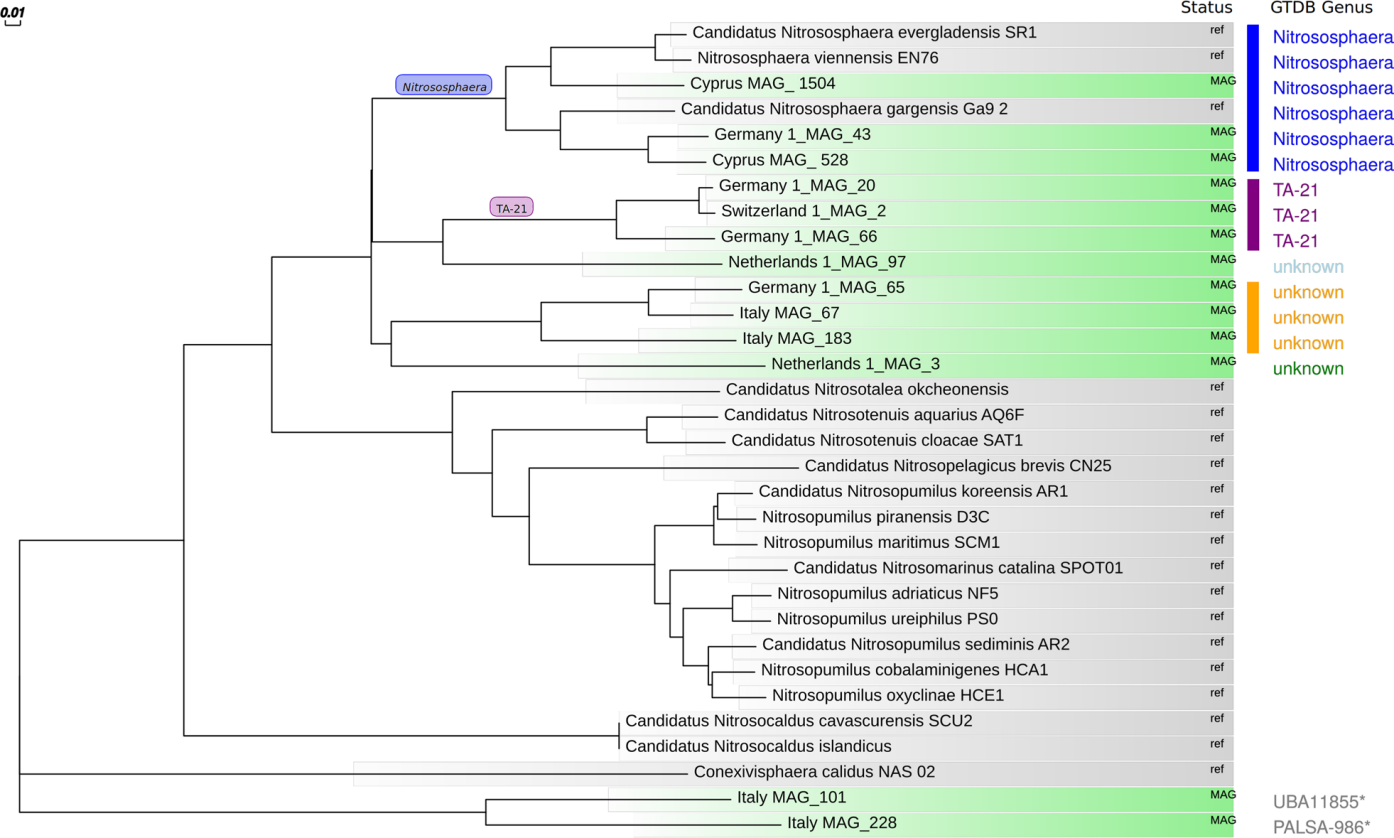
Phylogenetic tree showing the placement of *Thaumarchaeota* soil microbiome members represented by reconstructed MAGs (light green bars) relative to the complete reference genomes of the phylum *Thaumarchaeota* from the NCBI genome database (grey bars). The tree was built out of a core of 22 genes per genome. The core corresponds to 9271 amino acid residues per genome. Genus affiliations according to the GTDB classification are named in colored text (blue *Nitrososphaera*, purple *TA-21*, yellow: genus unknown but the clustering suggests a common genus). The phylogenetic analysis was performed within the EDGAR 3.0 platform [33]. The bar indicates one substitution per 100 positions. *UBA11855 and PALSA-986 belong to the *Thermoproteota* phylum according to the GTDB taxonomy [19]. In the NCBI taxonomy these genera are not named and were classified to belong to the phylum *Bathyarchaeota*

**Table 3:** Summary of Metagenomically Assembled Genomes (MAGs) assigned to the phylum *Thermoproteota/Thaumarchaeota* compiled from metagenomic sequences of European agricultural soils

MAG	Equivalent in [4]	Genus (GTDB)	Last common ancestor (GTDB)	Completeness	Contamination
Germany_1_MAG_43	MAG_04	*Nitrososphaera*		76.21	5.97
Germany_1_MAG_65	MAG_02	–	Nitrososphaeraceae (F)	60.02	7.93
Germany_1_MAG_66	MAG_03	TA-21		91.75	8.74
Germany_1_MAG_20	MAG_01	TA-21		95.15	7.08
Switzerland_1_MAG_2		TA-21		95.95	1.53
Netherlands_1_MAG_97		–	Nitrososphaeraceae (F)	50.24	0
Netherlands_1_MAG_3		–	Nitrososphaeraceae (F)	86.73	2.27
Italy_MAG_183		–	Nitrososphaeraceae (F)	99.03	0
Italy_MAG_67		–	Nitrososphaeraceae (F)	97.09	1.94
Italy_MAG_228		PALSA-986*		84.75	7.48
Italy_MAG_101		UBA11855*		89.72	10.28
Cyprus_MAG_528		*Nitrososphaera*		60.19	2.17
Cyprus_MAG_1504		*Nitrososphaera*		72.49	4.37

* *Bathyarchaeota* in NCBI taxonomy

**Fig. 6:**
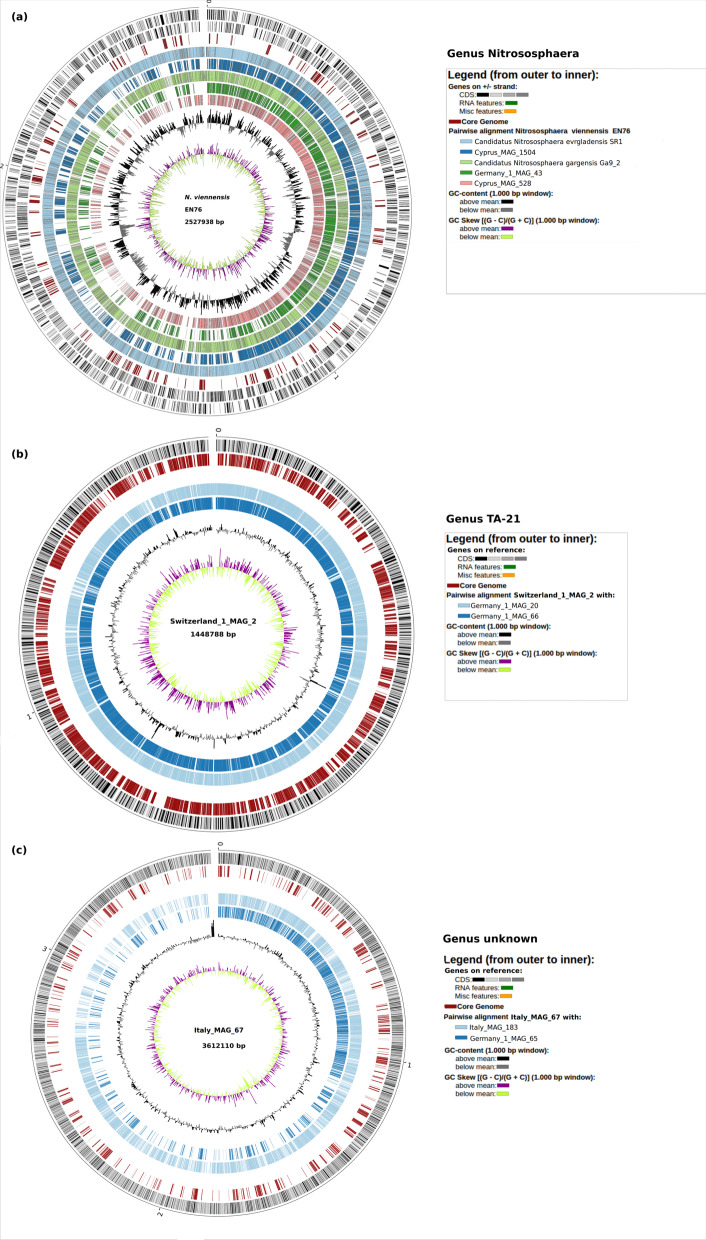
Circular representation of the similarity between genomes clustering closely in the phylogenetic tree (Fig. 5). Orthologous genes of the analyzed MAGs are plotted relative to their position in the respective reference genomes (outermost rings). Core genes of the analyzed genomes are plotted in red. The individual concentric rings represent the pairwise core genome with the reference. (**a**) Genus *Nitrososphaera*. Reference sequence is the genome of the NCBI reference genome *N. viennensis* EN76 (NCBI:txid926571, Accession No. NZ_CP007536). (**b**) Genus TA-21 according to GTDB (https://gtdb.ecogenomic.org/) (reference sequence is the MAG Switzerland_1_MAG_2 of this study). (**c**) Unknown Genus (reference sequence is the MAG Italy_MAG_67). The innermost circles rpresent GC skew plots (purple above mean, light green below mean) and GC content plots showing deviations from the average (black and gray). The circular plots were generated with BioCircos within EDGAR3 [33]

The binning of metagenomically assembled contigs to metagenomically assembled genomes (MAGs) yielded in total 2187 MAGs. We further subjected the MAGs to a taxonomic classification, revealing the successful binning of 13 *Thaumarchaeota*/*Thermoproteota* MAGs fulfilling our quality standards (Table 3). Twelve of the MAGs were classified as members of the family *Nitrososphaeraceae*, two MAGs, namely Italy_MAG_228 and Italy_MAG_101 were assigned to genera belonging to the GTDB taxonomy phylum *Thermoproteota*. Those genera are not named in the NCBI taxonomy and are most similar to the Candidatus *Bathyarchaeota* phylum. Figure 5 shows the placement of the 13 retrieved MAGs in a phylogenetic tree relative to available complete reference genomes for the phylum *Thaumarchaeota* (NCBI), based on 22 core genes. The *Nitrososphaeraceae* MAGs are closer to the *Nitrososphaera* genomes than to other thaumarchaeotal genera from different families and Italy_MAG_228 and Italy_MAG_101 are outliers. Further, the phyolgenetic tree supports the taxonomic assignment (Table 3), as all *Nitrososphaera*-assigned MAGs aggregate in one cluster (blue box in Fig. 5) and the MAGs assigned to the genus TA-21 form a separate distinct cluster (red box in Fig. 5). Interestingly, Switzerland_1_MAG_2 and Germany_1_MAG_20 cluster very tightly within this TA-21 cluster. Their similarity is further supported by their pairwise median Average Amino Acid Identity (AAI) of more than 99%. We observed a third cluster (yellow), which might represent a new *Nitrososphaeraceae* genus. Based on the observed genus clusters, we visualized the genomes in circular representations of the pairwise alignments of orthologous genes in the *Nitrososphaera* MAGs with the reference genome *Nitrososphaera viennensis* EN76 (Fig. 6a), the TA-21 MAGs with the most complete TA-21 MAG Switzerland_1_MAG_2 (Fig. 6b) and accordingly for MAGs in the potential genus cluster with Italy_MAG_67 (Fig. 6c).

**Fig. 7:**
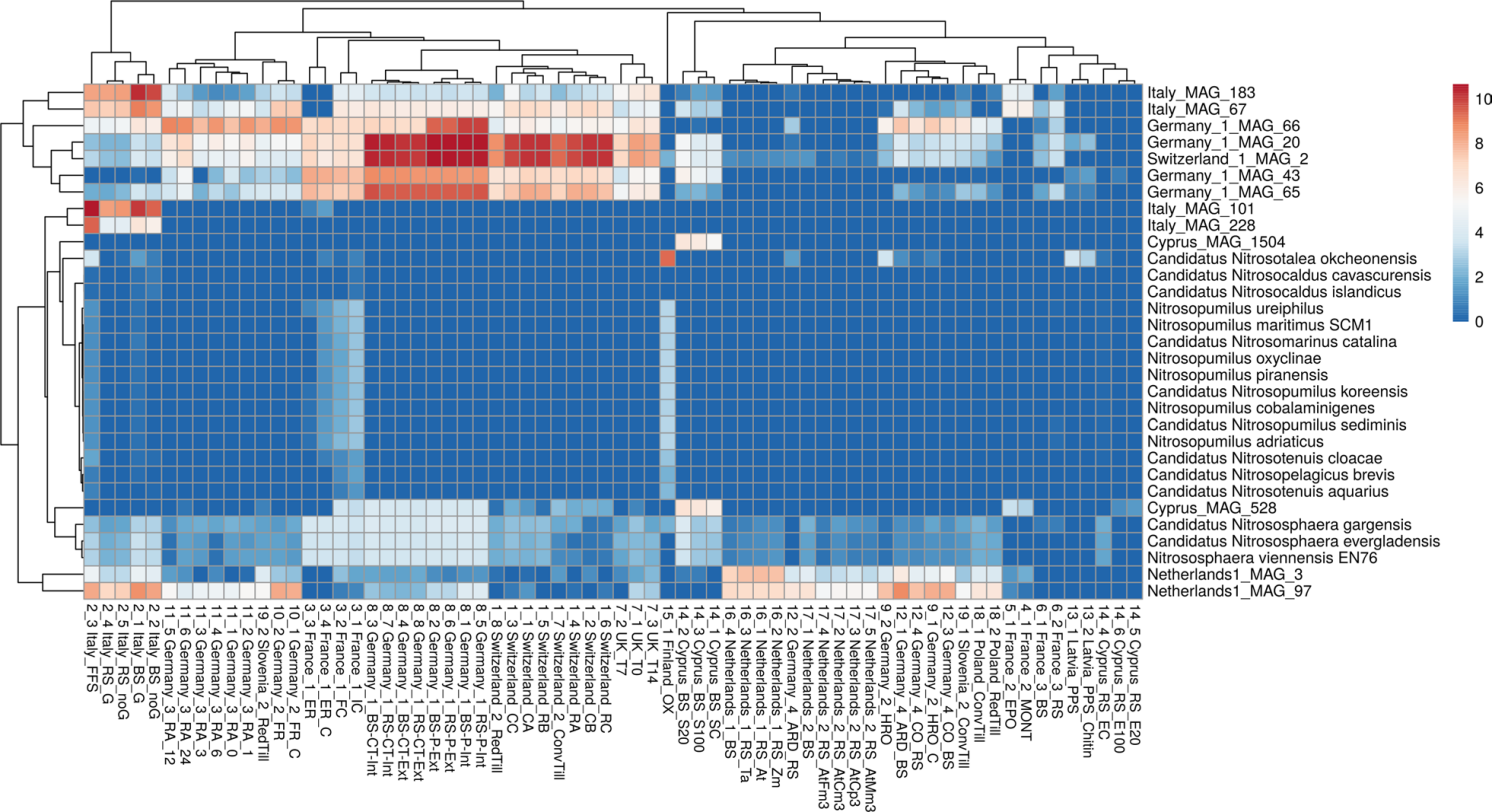
Occurrence heatmap of *Thaumarchaeota* complete reference genomes and MAGs reconstructed from the selected agricultural soil microbiomes, as determined by fragment recruitments. The scale (ln(x)-transformed) represents the abundance normalized to 1 M reads. With a maximum of 42528.17 normalised abundance (4.25% relative abundance), the ln(x)-scaled maximum value is at 10.66. The color scale ranges from blue (no abundance) to yellow (medium abundance) to red (high abundance)

Members of the genus TA-21 seem to be relevant in almost all of the soils studied (Fig. 7). Therefore, exemplarily for the reconstructed MAGs, genome mining for a metabolic reconstruction was applied to Switzerland_1_MAG_2.

#### Metabolic reconstruction of Switzerland_1_MAG_2

Switzerland_1_MAG_2 reconstructed from the metagenomes obtained within the Switzerland_1 study was assigned to the genus TA-21 of the family *Nitrososphaeraceae*. Currently, GTDB lists six species representatives for the genus TA-21 which were assembled from metagenomes from a temperate grassland biome [42] or a river sediment (unpublished), respectively. Switzerland_1_MAG_2 is almost complete (96%) and features a low contamination rate (1.5%) and 1,632 predicted genes (Fig. 6). *Carbohydrate metabolism* Concerning its carbohydrate metabolism, genome mining revealed that Switzerland_1_MAG_2 encodes complete KEGG modules for gluconeogenesis and the non-oxidative pentose phosphate pathway for transformation of C4, C5, C6 and a C7 sugar into each other. Moreover, the citrate cycle is almost complete (only one gene for a citrate cycle enzyme has not been identified) and the MAG has the potential to convert propanoate to succinate *via* methyl-malonyl-CoA (propanoate metabolism). The volatile fatty acid (VFA) propanoate is an intermediate metabolite in biomass decomposition. Further, twelve of sixteen enzymes of the carbon dioxide (CO$_2$) fixation pathway (3-hydroxypropionate/4-hydroxybutyrate cycle, KEGG module M00375) were predicted to be encoded in Switzerland_1_MAG_2. Genes for the two carboxylation key-enzymes acetyl-CoA carboxylase (EC 6.4.1.2), and propionyl-CoA carboxylase (EC 6.4.1.3) and 4-hydroxybutanoyl-CoA dehydratase (EC 4.2.1.120) were identified in the genome. Accordingly, the species represented by Switzerland_1_MAG_2 is predicted to fix CO$_2$ for the synthesis of succinyl-CoA which probably is the primary carbon fixation product [43].

*Pyruvate and mevalonate metabolism*

The enzymes malate dehydrogenase (malic enzyme, EC 1.1.1.38 and EC 1.1.1.37) and pyruvate dehydrogenase have functions in pyruvate metabolism for pyruvate interconversion to malate and further to oxaloacetate or to acetate, respectively. Phosphoenol-pyruvate carboxykinase (EC 4.1.1.49) catalyzes the reaction from oxaloacetate to phosphoenol-pyruvate that may enter the gluconeogenesis pathway. Switzerland_1_MAG_2 encodes four enzymes of the mannose metabolism that were predicted to catalyze the reactions from mannose-6-phosphate to mannosylglycerate *via* two intermediates. Mannosylglycerate is known as a compatible solute which could imply an adaptive advantage in soil under certain conditions. Interestingly, Switzerland_1_MAG_2 may be able to convert acetyl-CoA *via* mevalonate to isopentenyl-pyrophosphate (mevalonate pathway of the terpenoid backbone biosynthesis). All but one enzyme of the mevalonate pathway are encoded in Switzerland_1_MAG_2. Isopentenyl-PP may be further converted to geranyl-PP, farnesyl-PP and geranyl-geranyl-PP. From the latter metabolite, gibberellins (diterpenoid biosynthesis) representing phytohormones may be synthesized. Therefore, a beneficial effect by Switzerland_1_MAG_2 on plant growth is conceivable.

*Nitrogen metabolism*

Concerning its nitrogen metabolism, Switzerland_1_MAG_2 encodes an ammonia monooxygenase (AMO) for ammonia oxidation to hydroxylamine. The further metabolism of hydroxylamine is currently being investigated. However, since Switzerland_1_MAG_2 encodes a nitrite reductase (NO-forming, NirK), nitric oxide (NO) may be formed which is known as a signaling molecule in plants. It may affect root growth and proliferation of root cells also involving the phytohormone auxin [44]. This is a further indication that Switzerland_1_MAG_2 may affect plant physiology. Since Switzerland_1_MAG_2 also possesses genes for ureases, these enzymes may deliver ammonium for the AMO-catalyzed reaction and carbon dioxide entering the CO$_2$ fixation pathway (see above). Glutamate dehydrogenase (EC 1.4.1.3) and glutamine synthetase (EC 6.3.1.2) complement the nitrogen metabolism of Switzerland_1_MAG_2.

*Carbohydrate-active enzymes*

A dbCAN analysis (web server and database for automated carbohydrate-active enzyme annotation) revealed that Switzerland_1_MAG_2 encodes several carbohydrate-active enzymes. Among these are enzymes belonging to the glycosyltransferase families GT2, GT4, GT55, GT66, and GT83, the glycoside hydrolase families GH5, GH109, GH130, and GH133. Further dbCAN hits represent enzymes of the carbohydrate esterase family CE4 and the carbohydrate-binding module family CBM32. Two of the identified GT family enzymes are homologous to enzymes encoded in two *N. viennensis* EN76 gene clusters predicted to be involved in exopolysaccharide (EPS) production, modification and/or N-glycosylation [45]. EPS-production is believed to be of importance for formation and stabilization of soil micro-aggregates and biofilms. Moreover, EPS protects its host from dehydration and may at least to some extent retain water in the system. Therefore, EPS-production facilitates survival and competitiveness of microorganisms in soil. However, confirmation of EPS-production for Switzerland_1_MAG_2 will only be possible when a corresponding isolate is available.

#### Genetic potential of other *Thaumarchaeota* MAGs

Germany_1_MAG_66, Germany_1_MAG_20 and France_1_MAG_1 were also assigned to the genus TA-21 (*Nitrososphaeraceae*). While Germany_1_MAG_66 and Germany_1_MAG_20 were also predicted to feature a high completeness (with slightly higher contamination values than Switzerland_1_MAG_2), France_1_MAG_1 in contrast is only 41.6% complete and has a contamination rate of 5.3%. Nevertheless, this MAG seems to encode the metabolic features described for Switzerland_1_MAG_2, however less complete. Germany_1_MAG_20 encodes a putative polyketide cyclase. Polyketides are structurally diverse and biologically active secondary metabolites; some show antibiotic or antifungal characteristics. In a comparative metatranscriptome analysis of wheat rhizosphere microbiomes, a polyketide cyclase has been shown to be differentially expressed in suppressive soil samples [46]. Concerning the beneficial potential regarding plant growth promotion of the reconstructed MAGs, we searched for genetic determinants of PGP. All of the MAGs were predicted to encode at least one alkaline phosphatase (AlPase), which is known in the plant-growth beneficial context because the enzyme is involved in solubilization of compounds containing phosphorus [47]. Most thaumarchaeotal MAGs possess genes encoding enzymes associated with the biosynthesis of auxins, e.g. anthranilate phosphoribosyltransferase (*trpD*) and anthranilate synthase [48, 49]. These enzymes are involved in formation of an precursor of the main natural plant auxin indole-3-acetic acid (IAA) [49]. Further, the gene *ribE* encoding riboflavin synthase was predicted, riboflavin is associated with stimulation of plant growth [50].

Germany_1_MAG_65, Italy_MAG_67 and Italy_MAG_183 represent a so far unknown *Nitrososphaeraceae* genus (see Fig. 5). Both MAGs from the Italian study feature a high completeness (above 97%) and low contamination rates (below 2%) whereas Germany_1_MAG_65 only has a completeness of 60% (Tab. 3). Therefore, metabolic reconstruction was focused on the two Italian *Nitrososphaeraceae* MAGs. Similar to Switzerland_1_MAG_2, both Italian MAGs also encode the complete KEGG module for gluconeogenesis, and almost complete (one block missing) modules of the non-oxidative pentose phosphate pathway and the citrate cycle. Likewise, the 3-hydroxypropionate/4-hydroxybutyrate carbon dioxide fixation pathway is almost completely encoded in these MAGs and they were predicted to be able to convert mannose-6-phosphate to mannosylglycerate. Moreover, both MAGs possess the mevalonate pathway and predictively oxidize ammonia to hydroxylamine.

In comparison to the pangenomes of members belonging to the genera *Nitrososphaera* and TA-21, 257 unique genes were identified in the core genome of Italy_MAG_67 and Italy_MAG_183. However, 248 of these unique genes were annotated to encode hypothetical proteins. Only nine unique genes received a functional annotation. Their predicted gene products, *i.a.*, represent a virginiamycin B lyase, a 4-carboxymuconolactone decarboxylase and an alkanesulfonate monooxygenase. Virginiamycin is a macrolide antibiotic of the streptogramin class. Therefore, resistance to type B streptogramin antibiotics might be common to the new genus, since the presence of a virginiamycin B lyase suggests the ability to cleave this cyclic antibiotic [51]. Moreover, the gentic potential to produce 4-carboxymuconolactone decarboxylase suggests the ability to degrade aromatic compounds [52]. Alkanesulfonate monooxygenase is known to be involved in sulfate assimilation in bacteria [53]. The ability to utilize sulfur-containing molecules from the environment could be an advantageous feature, since sulfur is critical for the synthesis of amino acids and enzyme cofactors.

Based on the identified unique genes with predicted functions, only preliminary assumptions can be made about the specific features applying to the new genus. However, members of the new genus share characteristic traits such as the ability to fix carbon dioxide and oxidize ammonia with the genera *Nitrososphaera* [45] and TA-21. These features may therefore be considered to represent common characteristics of all previously known species of the family *Nitrososphaeraceae*.

Further analyses addressed the abundances of the reconstructed *Thaumarchaeota* MAGs in soil, in order to check in which agricultural soils next to their original soils these microorganisms might contribute to important soil functions.

#### Occurrence of reconstructed *Nitrososphaeraceae* MAGs

To evaluate the indigenous occurrence in other European soils of *Thaumarchaeota* reference genomes and the *Thaumarchaeota*/*Thermoproteota* MAGs which were derived from European agricultural soils, metagenome fragment recruitments were performed. As expected, the *Thaumarchaeota* MAGs were mostly identified in their original soil environment (Fig. 7). In the other soils, they are limited domiciled. Strikingly, MAGs and reference genomes belonging to the *Nitrososphaeraceae* family were most abundant in the European agricultural soils. Members of other *Thaumarchaota* families were prevalent in the soil micobiome from Finland and France_1, e.g. the Finnish soil showed a high abundance of the *Nitrosotalea* reference genome. In the sample Finland_OX, the sample collection depth was significantly higher (75 cm) than in all other samples. Thus, those *Thaumarchaeota* species might be well adapted to low availability of oxygen and low pH (3.7). In France_1 soil samples, *Nitrosotenuis*, *Nitrosopumilus*, and *Nitrosopelagicus* and additionally *Nitrosocaldus* genomes were identified. Interestingly, they seem to be sensitive to biostimulants applied in this study, since they were more prevalent in the initial and final control compared to the samples treated with biostimulants (France_1_ER: treated with a phenolics-based root exudate inductor, France_1_ER_C treated with the former and additionally a microbial product based on *Pseudomonas fluorescens* and *Trichoderma harzianum*).

## Conclusion

*Thaumarchaeota* members were detected in all agricultural soil metagenomes analyzed in this meta-study. Although they are most abundant in the highly fertile loess-chernozem soil from Germany (Germany_1), *Thaumarchaeota* members seem to be of importance in all of the other soils. The fact, that *Thaumarchaeota* MAGs are among the MAGs that could be reconstructed from soil metagenome sequencing data, highlights their importance for agricultural soils. Notably, they mostly belong to the *Nitrososphaeraceae* family. They might represent soil health ameliorating candidates since they were predicted to fix carbon dioxide (CO$_2$), contribute to the soil nitrogen cycle by oxidation of ammonia and may produce precursors for phytohormones. Further, due to their EPS-producing potential, the *Thaumarchaeota* MAGs may contribute to soil micro-aggregate stabilization. An often mentioned goal of current research focussing on PGP microorganisms (PGPMs) as soil additives is the safe and sustainable use of PGPMs as biological fertilizers. This may decrease the need for detrimental fertilizers and agrochemicals for the defence against phytopathogenic microorganisms, and could help to biologically control crop diseases.

Our results will be important for further studies elaborating the contribution of *Thaumarchaeota* to the high fertility of Chernozem soils (’Black soils’). Of special interest should be, how *Thaumarchaeota* abundance can be put into context regarding soil productivity in terms of crop yield.

Ultimately, to control between-study heterogeneity and to more elaborately assess the environmental factors that contribute to a healthy soil microbiome, more primary research is still needed. The metadata table we provided for the soil locations studied here can serve as a framework for metadata collection in future studies on soil metagenomes. Sustainable and consistent metadata compilation remains a challenge. Interpretation of data in meta studies ultimately relies on the recorded metadata of the primary studies. Recent attempts and initiatives such as for example the German National Research Data Infrastructure (NFDI) tackle the challenge of harmonized and centralized collection of research data. The ‘Land Use/Cover Area frame statistical Survey’ (LUCAS Soil) provides a regular and standardized collection of soil data for the entire territory of the European Union (EU), addressing all major land cover types simultaneously, in a single sampling period [54]. Metagenome sequencing data from LUCAS agricultural soils is a valuable resource for further analysing the role of *Thaumarchaeota*. Our meta study highlights the necessity to unify metadata collection for sequenced soil microbiomes in order to enable the discovery of correlations and interrelationships by networking open data.

## Supplementary Information

**Additional file 1**. Scopes of the primary studies: Scopes and details of the primary studies incorporated into this meta study.

**Additional file 2**. Metadata Table: Detailed metadata table of the primary studies.

**Additional file 3**. Distribution of Thaumarchaeota subtaxa per soil location: Sankey diagrams of the Thaumarchaeota subtaxa distribution shown for all soil locations.

## Data Availability

The primary study’s data accession numbers are given in the material and methods section.

## Abbreviations

MAG: Metagenomically assembled genome
PGP: Plant-growth-promotion
PGPMs: Plant-growth-promoting microorganisms
EPS: Extracellular polymeric substances
SRA: Sequence read archive
ENA: European nucleotide archive
EMGB: Elastic metagenome browser
Gb: Gigabases
AAI: Average aminoacid identity
VFA: Volatile fatty acid
AMO: Ammonia monooxygenase
NO: Nitric oxide
CO$_2$: Carbon dioxide
