# Supplementary material for: Abundance, classification and genetic potential of Thaumarchaeota in metagenomes of European agricultural soils: a meta-analysis
Source: Environ Microbiome. 2023 Mar 30;18:26. doi: 10.1186/s40793-023-00479-9 (PMC10064710; doi:10.1186/s40793-023-00479-9)
Supplement: Supplementary file 1 — Additional file 1. Scopes of the primary studies: Scopes and details of the primary studies incorporated into this meta study. [file 40793_2023_479_MOESM1_ESM.pdf]

Additional File 1 – Scopes of the primary studies

1. Switzerland

This study (Project number PRJNA387672 in SRA) from Cania et al. was published 2019 [https://environmentalmicrobiome.biomedcentral.com/articles/10.1186/s40793-019-0341-7]. Object of the study is the microbial community of the Frick long-term field trial soil in Switzerland. The study demonstrates the influence of tillage on the bacterial potential to produce soil structure-stabilizing agents (exopolysaccharides and lipopolysaccharides) in the context of stable aggregate formation. The authors compared two tillage treatments sampled at three depths. For our metastudy we therefore combined the replicates into six treatments, resulting in the following data points:

1. Switzerland_CA: Soil from 0-10 cm depth, conventionally-tilled plot fertilized with slurry
2. Switzerland_CB: Soil from 10-20 cm depth, conventionally-tilled plot fertilized with slurry
3. Switzerland_CC: Soil from 20-50 cm depth, conventionally-tilled plot fertilized with slurry
4. Switzerland_RA: Soil from 0-10 cm depth, reduced-tilled plot fertilized with slurry
5. Switzerland_RB: Soil from 10-20 cm depth, reduced-tilled plot fertilized with slurry
6. Switzerland_RC Soil from 20-50 cm depth, reduced-tilled plot fertilized with slurry

2. Italy

The Italian soil stems from a rice paddy located in the valley of the River Po. Rice paddies are of importance for the food supply but are considered a major source of the greenhouse gas methane. The study by Zecchin et al. (PRJNA393632, accession named in paper PRJNA391190) was published 2017 and is dedicated to the investigation of native dissimilatory sulfate-reducing microbial populations and to characterize their metabolic properties. Therefore, the metagenomes were assembled from rice paddy soil that was used to grow rice in the presence and absence of gypsum ($CaSO_4 \cdot 2H_2O$). By this, the authors were able to identify Nitrospirae spp. which carry and express genes related to sulfate respiration. [https://aem.asm.org/content/84/5/e02224-17.full#sec-10]

Further metadata resource: https://www.sciencedirect.com/science/article/abs/pii/S0167880900002656

For our metastudy, we divided the available sequencing files therefore into five treatments:

2_1 Italy_BS_G	Bulk soil with gypsum
2_2 Italy_BS_noG	Bulk soil without gypsum
2_3 Italy_FFS	Freshly flooded soil
2_4 Italy_RS_G	Rhizosphere soil with gypsum
2_5 Italy_RS_noG	Rhizosphere soil without gypsum

3. France_1

Crovadore et al. analyzed the metagenomes of soil samples from an established perennial cropping system of asparagus treated with biostimulants located in southern france (study accession PRJNA378475) [https://mra.asm.org/content/ga/5/24/e00511-17.full.pdf]. By personal correspondence, further metadata of the field could be retrieved.

According to the described experiment, we divided for our meta study into four treatments:

1. France_1_IC Initial control
2. France_1_FC Final control
3. France_1_ER Treated with ExuRoot
4. France_1_ER_C Treated with ExuRoot and Cérès

4. & 5. France_2

To measure the impact of phages on soil bacterial communities and nitrogen availability, the authors set up a reciprocal transplant experiment using natural and sterilized soil incubated with different combinations of two soil microbial communities, challenged against native and non-native phage suspensions as well as against a cocktail of phage isolates (PRJNA550482). [https://microbiomejournal.biomedcentral.com/articles/10.1186/s40168-020-00822-z#Sec11]

By advice from the main author, we divided the samples into the soil origins:

2 Treatments:

4_1.France_2_MONT Montpellier
5_1.France_2_EPO Epoisses Dijon

6. France_3

In this study (PRJEB12917), the authors explored the consortia involved in polycyclic aromatic hydrocarbons (PAHs) degradation relevant for rhizoremediation of polluted soils. They found that taxonomically diverse co-occurring bacteria perform successive metabolic steps. Degradation of 13C-phenanthrene was monitored and fractions of 13-C-labeled DNA was compared in two conditions, bulk soil or planted with ryegrass. https://www.ncbi.nlm.nih.gov/pmc/articles/PMC6775975

Further metadata resource https://www.ncbi.nlm.nih.gov/pmc/articles/PMC2753067/

- Treatments:

1 France_3_BS Bare soil
2 France_3_RS Ryegrass-vegetated soil

7. United_Kingdom

Grassland soil was a subject of this study (PRJNA390514) analysing the methanethiol-dependent dimethylsulfide production in soil environments. Based on the supplementary information we divided the samples into three timepoints. https://www.nature.com/articles/ismej2017105

- T0 Rhizosphere soil
- T7 Bulk soil
- T14 Bulk soil

8. Germany_1

In our previous study (PRJEB31111), agricultural soil from a long term experiment at Bernburg (Saxony-Anhalt, Germany, highly fertile Chernozem soil) was analyzed regarding differences in microbial community composition between bulk soil and root-influenced soil, tillage (reduced vs. conventional) and nitrogen fertilization (intensive vs. extensive). The indigenous soil microbiome was characterized at the taxonomic

and functional level focusing on the identification of potential plant-beneficial, plant-growth-promoting and biocontrol determinants. Moreover, soil metagenome sequences were assembled and taxonomically binned to yield Metagenomically Assembled Genomes (MAGs) representing abundant soil microbiome members. MAGs were functionally profiled by genome-based metabolic reconstruction to identify putative new plant-growth-promoting or plant-beneficial bacteria. Soil management practices affect the abundance of particular MAGs. Importance of the phylum Thaumarchaeota for the analyzed microbiome is corroborated by the fact that the four corresponding MAGs were predicted to oxidize ammonia (nitrification), thus contributing to the cycling of nitrogen, and in addition are most probably able to fix carbon dioxide. Moreover, Thaumarchaeota and several bacterial MAGs also possess genes with predicted functions in plant–growth–promotion. https://www.mdpi.com/2073-4425/10/6/424/htm

8 Treatments:

Germany_1_BS-P-Int Bulk soil, ploughed with standard nitrogen fertilization and use of pesticides
Germany_1_BS-P-Ext Bulk soil, ploughed with reduced (50%) nitrogen fertilization
Germany_1_BS-CT-IntBulk soil, cultivator treatment with standard nitrogen fertilization and use of pesticides
Germany_1_BS-CT-Ext Bulk soil, cultivator treatment with reduced (50%) nitrogen fertilization
Germany_1_RS-P-Int Root-affected soil, ploughed with standard nitrogen fertilization and use of pesticides
Germany_1_RS-P-Ext Root-affected soil, ploughed with reduced (50%) nitrogen fertilization
Germany_1_RS-CT-Int Root-affected soil, cultivator treatment with standard nitrogen fertilization and use of pesticides
Germany_1_RS-CT-Ext Root-affected soil, cultivator treatment with reduced (50%) nitrogen fertilization

9. & 10. Germany_2

in this study (PRJNA385596, https://sfamjournals.onlinelibrary.wiley.com/doi/10.1111/1758-2229.12651) microbial communities from agricultural soils from two long term field trials located in Freising (Bavaria) and Rostock (Mecklenburg Western-Pomerania), Germany, were used for studying bacterial potentials for uptake, solubilization and mineralization of extracellular phosphorus under different fertilization regimes. We separated the data into the four treatments:

9_1	Germany_2_HRO_C	HRO_autumn+spring_compost
9_2	Germany_2_HRO	HRO_autumn+spring_no_compost
10_1	Germany_2_FR_C	FR_autumn+spring_compost
10_2	Germany_2_FR	FR_autumn+spring_no_compost

11. Germany_3

The reclamation process of a coal mining site for agricultural use was monitored in this study (PRJNA557612) [https://www.ncbi.nlm.nih.gov/pmc/articles/PMC7017822/]. Aim of the study was to analyse potential EPS and LPS producing bacteria which contribute to soil aggregation and structure stabilization during soil formation, as affected by a management regime.
Further metadata resource: Philap et al. 2019
6 Treatments:

Germany_3_RA_0	Reclamation_age_0years
Germany_3_RA_1	Reclamation_age_1years
Germany_3_RA_3	Reclamation_age_3years
Germany_3_RA_6	Reclamation_age_6years
Germany_3_RA_12	Reclamation_age_12years
Germany_3_RA_24	Reclamation_age_24years

12. Germany_4

Differences on the functional potential of microbial communities present in the rhizosphere of apple plants grown in apple replant disease affected and non-affected soils (long-term field trail located in Ellerhoop, Schleswig-Holstein, Germany) was the scope of the study Germany_4 (PRJNA532820) [https://environmentalmicrobiome.biomedcentral.com/articles/10.1186/s40793-019-0346-2]

4 Treatments:

Germany_4_ARD_BS	Apple replant disease soil (replant), Bulk soil
Germany_4_ARD_RS	Apple replant disease soil (replant), Rhizosphere sc
Germany_4_CO_BS	Virgin soil (non replant), Bulk soil
Germany_4_CO_RS	Virgin soil (non replant), Rhizosphere soil

13. Belgium

- In this study, the influence of chitin addition on the lettuce rhizosphere microbiome was studied; Lettuce plants were grown in potting soil from Latvia: http://www.humibox.com/products/growing-media/peltracom . The results showed that chitin addition increases chitin-catabolic enzymes, bacterial ammonium oxidizing and siderophore genes. Occurrence of increased ammonium oxidizing bacteria, *Nitrosospira*, and *amoA* genes results in an elevated concentration of plant-available nitrate. In addition, the increase in chitinase and siderophore genes may have stimulated the plant's systemic resistance. (PRJEB15448) https://www.nature.com/articles/s41598-019-46106-x
- Treatments
-

Belgium_Latvia_PPS	PeatPottingSoil
Belgium_Latvia_PPS_(	PeatPottingSoil with Chitin-amendmend

•

14. Cyprus

- This study examined the soil and *Lactuca sativa* resistome under exposure of antibiotics through irrigation water. (PRJEB35612) https://www.sciencedirect.com/science/article/pii/S0304389420311973?via%3Dihub
- Treatments

Cyprus_BS_SC	Bulk soil watered with 0 µg antibiotics
Cyprus_BS_S20	Bulk soil watered with 20 µg antibiotics
Cyprus_BS_S100	Bulk soil watered with 100 µg antibiotics
Cyprus_RS_EC	Rhizosphere soil watered with 0 µg antibiotics
Cyprus_RS_E20	Rhizosphere soil watered with 20 µg antibiotics
Cyprus_RS_E100	Rhizosphere soil watered with 100 µg antibiotics

•

15. Finland

- https://www.nature.com/articles/s41597-019-0222-3
- Soil microbial communities from Risofladan, Vaasa, Finland : boreal potential and actual acid sulfate soil materials
- Treatments:
-

Finland_OX	oxidized soil layer (approximately 75–140 cm depth	•
Finland_TR	transition soil layer	
Finland_UN	un-oxidized soil layer	

16. Netherlands_1

- https://www.ncbi.nlm.nih.gov/pmc/articles/PMC6908837/
- **Genome-Resolved Proteomic Stable Isotope Probing of Soil Microbial Communities Using 13CO2 and 13C-Methanol:** Here, proteomic SIP was combined with targeted metagenomic binning to reconstruct metagenome-assembled genomes (MAGs) of the microorganisms producing labeled proteins. This approach was used to track carbon flows from 13CO2 to the rhizosphere communities of *Zea mays*, *Triticum aestivum*, and *Arabidopsis thaliana*. (PRJNA488251)
- 4 Treatments:
- Netherlands_1_RS_At Rhizosphere community A. thaliana
- Netherlands_1_RS_Zm Rhizosphere community Z. mays
- Netherlands_1_RS_Ta Rhizosphere community T. aestivum
- Netherlands_1_BS Metagenomes of initial bulk soil samples (grassland)

17. Netherlands_2

- https://www.pnas.org/content/115/22/e5213 , https://nph.onlinelibrary.wiley.com/doi/10.1111/nph.15798
- Description from ENA: Metagenome of bulk soil and root samples of different Arabidopsis wild type and mutant plants that have different exudation patterns (PRJNA435676)
- Further metadata resource: https://www.nature.com/articles/s41396-018-0093-1.pdf?origin=ppub
- 5 Treatments:
- Netherlands_2_BS Bulk soil
- Netherlands_2_RS_AtCm3 Col_minus
- Netherlands_2_RS_AtCp3 Col_plus
- Netherlands_2_RS_AtFm3 f6h1_minus
- Netherlands_2_RS_AtMm3 myb72_minus

1., 18. & 19. Poland, Slovenia and Switzerland

- PRJNA555481
- https://www.ncbi.nlm.nih.gov/pmc/articles/PMC7154075/
- Metagenomes of two tillage treatments from three agricultural trials – Two tillage treatments sampled from three agricultural field trials in Frick (Switzerland, same as in the first study), Moškanjci (Slovenia) and Juchowo (Poland). The aim was to study the influence of different tillage intensities on microbial community structure and functionality in soils with different properties. Functional analysis focused on genes catalyzing the biosynthesis and export of exo- and lipopolysaccharides.
- **Site-Specific Conditions Change the Response of Bacterial Producers of Soil Structure-Stabilizing Agents Such as Exopolysaccharides and Lipopolysaccharides to Tillage Intensity:**
- 6 Treatments:
- 1_7 Switzerland_2_ConvTill Conventional tillage
- 1_8 Switzerland_2_RedTill Reduced tillage

- 18_1 Poland_ConvTill Conventional tillage
- 18_2 Poland_RedTill Reduced tillage
- 19_1 Slovenia_2_ConvTill Conventional tillage
- 19_2 Slovenia_2_RedTill Reduced tillage

20. Denmark (excluded, since enriched microbial consortium and no soil metagenome)

- https://www.sciencedirect.com/science/article/abs/pii/S0048969720368121
- Further metadata resource: https://doi.org/10.1016/j.biortech.2018.09.006
- Metagenomic analysis of a keratin-degrading bacterial consortium, obtained by selective enrichment from a soil sample. (PRJEB38982)
- 1 Treatment
  Denmark_Slovenia_KMC Keratinolytic microbial consortium from River bank in Slovenia
