# Supplementary material for: Abundance, classification and genetic potential of Thaumarchaeota in metagenomes of European agricultural soils: a meta-analysis
Source: Environ Microbiome. 2023 Mar 30;18:26. doi: 10.1186/s40793-023-00479-9 (PMC10064710; doi:10.1186/s40793-023-00479-9)
Supplement: Supplementary file 2 — Additional file 2. Metadata Table: Detailed metadata table of the primary studies. [file 40793_2023_479_MOESM2_ESM.pdf]

Table 1: Table: Selected studies of soil microbiomes with agricultural context and availability of metadata. Asteriks for metadata that were derived with resources other than the original primary study, *e.g.* personal communication or secondary study.

Loc ID	Name	Crop	Compartment	Tillage	Collection depth	Temp. in C	Soil type	pH	Soil texture	Percent sand	Percent silt	Percent clay
1_1	Switzerland_CA	Green manure	Bulk soil	Conventional	10 cm	8.9	Stagnic Eutric Cambisol	7.1	Clayey	22	33	45
1_2	Switzerland_CB	Green manure	Bulk soil	Conventional	20 cm	8.9	Stagnic Eutric Cambisol	7.1	Clayey	22	33	45
1_3	Switzerland_CC	Green manure	Bulk soil	Conventional	50 cm	8.9	Stagnic Eutric Cambisol	7.1	Clayey	22	33	45
1_4	Switzerland_RA	Green manure	Bulk soil	Reduced	10 cm	8.9	Stagnic Eutric Cambisol	7.1	Clayey	22	33	45
1_5	Switzerland_RB	Green manure	Bulk soil	Reduced	20 cm	8.9	Stagnic Eutric Cambisol	7.1	Clayey	22	33	45
1_6	Switzerland_RC	Green manure	Bulk soil	Reduced	50 cm	8.9	Stagnic Eutric Cambisol	7.1	Clayey	22	33	45
2_1	Italy_BS_G	Rice	Bulk soil	NA	10 cm	NA	Cambisol Sandy Loam*	6*	Sandy loam*	60*	25*	12*
2_2	Italy_BS_noG	Rice	Bulk soil	NA	10 cm	NA	Cambisol Sandy Loam*	6*	Sandy loam*	60*	25*	12*
2_3	Italy_FFS	Rice	Bulk soil	NA	NA	NA	Cambisol Sandy Loam*	6*	Sandy loam*	60*	25*	12*
2_4	Italy_RS_G	Rice	Rhizosphere soil	NA	3 cm	NA	Cambisol Sandy Loam*	6*	Sandy loam*	60*	25*	12*
2_5	Italy_RS_noG	Rice	Rhizosphere soil	NA	3 cm	NA	Cambisol Sandy Loam*	6*	Sandy loam*	60*	25*	12*
3_1	France_1_IC	Asparagus	Rhizosphere soil	NA	NA	NA	NA	NA	Sandy and Silty	NA	NA	NA
3_2	France_1_FC	Asparagus	Rhizosphere soil	NA	NA	NA	NA	NA	Sandy and Silty	NA	NA	NA
3_3	France_1_ER	Asparagus	Rhizosphere soil	NA	NA	NA	NA	NA	Sandy and Silty	NA	NA	NA
3_4	France_1_ER_C	Asparagus	Rhizosphere soil	NA	NA	NA	NA	NA	Sandy and Silty	NA	NA	NA
4_1	France_2_MONT	Wheat*	Bulk soil*	NA	NA	NA	NA	8.6	Clay loam	34.6	35.2	28.8
5_1	France_2_EPO	Wheat*	Bulk soil*	NA	NA	NA	NA	5.6	Silty clay	6.2	51.9	41.9
6_1	France_3_BS	Bulk soil	Bulk soil	NA	NA	NA	NA	7.25*	Sandy loam*	65.8*	22.1*	12.1*
6_2	France_3_RS	Green manure	Rhizosphere soil	NA	NA	NA	NA	7.25*	Sandy loam*	65.8*	22.1*	12.1*
7_1	UK_T0	Green manure*	Bulk soil	NA	NA	NA	NA	6.7	Sandy*	NA	NA	NA
7_2	UK_T7	Bulk soil*	Bulk soil	NA	NA	NA	NA	6.7	Sandy*	NA	NA	NA
7_3	UK_T14	Bulk soil*	Bulk soil	NA	NA	NA	NA	6.7	Sandy*	NA	NA	NA
8_1	Germany_1_BS-P-Int	Wheat	Bulk soil	Conventional	25 cm	9.7	Loess chernozem over limestone	7.2	Silty loam	8	70	22
8_2	Germany_1_BS-P-Ext	Wheat	Bulk soil	Conventional	25 cm	9.7	Loess chernozem over limestone	7.2	Silty loam	8	70	22
8_3	Germany_1_BS-CT-Int	Wheat	Bulk soil	Reduced	25 cm	9.7	Loess chernozem over limestone	7.2	Silty loam	8	70	22
8_4	Germany_1_BS-CT-Ext	Wheat	Bulk soil	Reduced	25 cm	9.7	Loess chernozem over limestone	7.2	Silty loam	8	70	22
8_5	Germany_1_RS-P-Int	Lettuce	Rhizosphere soil	Conventional	25 cm	9.7	Loess chernozem over limestone	7.2	Silty loam	8	70	22
8_6	Germany_1_RS-P-Ext	Lettuce	Rhizosphere soil	Conventional	25 cm	9.7	Loess chernozem over limestone	7.2	Silty loam	8	70	22
8_7	Germany_1_RS-CT-Int	Lettuce	Rhizosphere soil	Reduced	25 cm	9.7	Loess chernozem over limestone	7.2	Silty loam	8	70	22
8_8	Germany_1_RS-CT-Ext	Lettuce	Rhizosphere soil	Reduced	25 cm	9.7	Loess chernozem over limestone	7.2	Silty loam	8	70	22
9_1	Germany_2_HRO_C	Maize*	Bulk soil*	Conventional	NA	8.1	Stagnic Cambisol (loamy sand)	6.3	Loamy sand	NA	NA	NA
9_2	Germany_2_HRO	Maize*	Bulk soil*	Conventional	NA	8.1	Stagnic Cambisol (loamy sand)	6.3	Loamy sand	NA	NA	NA
10_1	Germany_2_FR_C	Maize*	Bulk soil*	Conventional	NA	8.4	Cambisol Silty loam	6.35	Silty loam	NA	NA	NA
10_2	Germany_2_FR	Maize*	Bulk soil*	Conventional	NA	8.4	Cambisol Silty loam	6.35	Silty loam	NA	NA	NA
11_1	Germany_3_RA_0	Bulk soil	Bulk soil	None	5 cm	9.5	Haplic Luvisol	7.5	Silty clay loam	3.5	65.1	31.4
11_2	Germany_3_RA_1	Alfalfa	Bulk soil	None	5 cm	9.5	Haplic Luvisol	7.5	Silty clay loam	3.5	65.1	31.4
11_3	Germany_3_RA_3	Alfalfa	Bulk soil	None	5 cm	9.5	Haplic Luvisol	7.5	Silty clay loam	3.5	65.1	31.4
11_4	Germany_3_RA_6	Wheat	Bulk soil	Conventional	5 cm	9.5	Haplic Luvisol	7.5	Silty clay loam	3.5	65.1	31.4
11_5	Germany_3_RA_12	Maize	Bulk soil	Conventional	5 cm	9.5	Haplic Luvisol	7.5	Silty clay loam	3.5	65.1	31.4
11_6	Germany_3_RA_24	Wheat	Bulk soil	Conventional	5 cm	9.5	Haplic Luvisol	7.5	Silty clay loam	3.5	65.1	31.4
12_1	Germany_4_ARD_BS	Bulk soil	Bulk soil	NA	20 cm	9.7	Endostagnic Luvisol	5.7	Loamy sand	72.5	15	10
12_2	Germany_4_ARD_RS	Apple plants	Rhizosphere soil	NA	20 cm	9.7	Endostagnic Luvisol	5.7	Loamy sand	72.5	15	10
12_3	Germany_4_CO_BS	Bulk soil	Bulk soil	NA	20 cm	9.7	Endostagnic Luvisol	5.7	Loamy sand	72.5	15	10
12_4	Germany_4_CO_RS	Apple plants	Rhizosphere soil	NA	20 cm	9.7	Endostagnic Luvisol	5.7	Loamy sand	72.5	15	10
13_1	Belgium_Latvia_PPS	Lettuce	Rhizosphere soil	NA	NA	NA	Peat soil	6.23	Peat soil	NA	NA	NA
13_2	Belgium_Latvia_PPS_Chitin	Lettuce	Rhizosphere soil	NA	NA	NA	Peat soil	6.03	Peat soil	NA	NA	NA
14_1	Cyprus_BS_SC	Bulk soil	Bulk soil	NA	NA	NA	Sandy clay loam	8.6	Sandy clay loam	46.1	21.1	32.8
14_2	Cyprus_BS_S20	Bulk soil	Bulk soil	NA	NA	NA	Sandy clay loam	8.6	Sandy clay loam	46.1	21.1	32.8
14_3	Cyprus_BS_S100	Bulk soil	Bulk soil	NA	NA	NA	Sandy clay loam	8.6	Sandy clay loam	46.1	21.1	32.8
14_4	Cyprus_RS_EC	Lettuce	Rhizosphere soil	NA	NA	NA	Sandy clay loam	8.6	Sandy clay loam	46.1	21.1	32.8
14_5	Cyprus_RS_E20	Lettuce	Rhizosphere soil	NA	NA	NA	Sandy clay loam	8.6	Sandy clay loam	46.1	21.1	32.8
14_6	Cyprus_RS_E100	Lettuce	Rhizosphere soil	NA	NA	NA	Sandy clay loam	8.6	Sandy clay loam	46.1	21.1	32.8
15_1	Finland_OX	Bulk soil	Bulk soil	NA	75cm	NA	Holocene estuarine clays and silts	3.7	Clayey	NA	NA	44.5
15_2	Finland_TR	Bulk soil	Bulk soil	NA	140cm	NA	Holocene estuarine clays and silts	4.7	Clayey	NA	NA	44.5
15_3	Finland_UN	Bulk soil	Bulk soil	NA	190cm	NA	Holocene estuarine clays and silts	8.1	Clayey	NA	NA	44.5
16_1	Netherlands_1_RS_At	Arabidopsis thaliana	Rhizosphere soil	NA	10 cm	NA	Loamy sand soil	NA	Loamy sand	NA	NA	NA
16_2	Netherlands_1_RS_Zm	Maize	Rhizosphere soil	NA	10 cm	NA	Loamy sand soil	NA	Loamy sand	NA	NA	NA
16_3	Netherlands_1_RS_Ta	Wheat	Rhizosphere soil	NA	10 cm	NA	Loamy sand soil	NA	Loamy sand	NA	NA	NA
16_4	Netherlands_1_BS	Bulk soil	Bulk soil	NA	10 cm	NA	Loamy sand soil	NA	Loamy sand	NA	NA	NA
17_1	Netherlands_2_BS	Bulk soil	Bulk soil	NA	NA	NA	Gleyic placic podzol	5.4	Sandy	88	8	2
17_2	Netherlands_2_RS_AtCm3	Arabidopsis thaliana	Rhizosphere soil	NA	NA	NA	Gleyic placic podzol	5.4	Sandy	88	8	2
17_3	Netherlands_2_RS_AtCp3	Arabidopsis thaliana	Rhizosphere soil	NA	NA	NA	Gleyic placic podzol	5.4	Sandy	88	8	2
17_4	Netherlands_2_RS_AtFm3	Arabidopsis thaliana	Rhizosphere soil	NA	NA	NA	Gleyic placic podzol	5.4	Sandy	88	8	2
17_5	Netherlands_2_RS_AtMm3	Arabidopsis thaliana	Rhizosphere soil	NA	NA	NA	Gleyic placic podzol	5.4	Sandy	88	8	2
1_7	Switzerland_2_ConvTill	Green manure	Bulk soil	Conventional	10 cm	8.9	Stagnic Eutric Cambisol, clayey	7.33	Clayey	31.3	23.01	45.95
1_8	Switzerland_2_RedTill	Green manure	Bulk soil	Reduced	10 cm	8.9	Stagnic Eutric Cambisol, clayey	7.08	Clayey	26.95	26.03	47.02
18_1	Poland_ConvTill	Lupine*	Bulk soil	Conventional	10 cm	8.5	Haplic Arenosol, sandy	6.3	Sandy	89.92	7.04	3.04
18_2	Poland_RedTill	Lupine*	Bulk soil	Reduced	10 cm	8.5	Haplic Arenosol, sandy	6.2	Sandy	92.38	5.83	1.79
19_1	Slovenia_2_ConvTill	Green manure	Bulk soil	Conventional	10 cm	10.6	Skeletic Eutric Cambisol, loamy	6.43	Loamy	37.6	43.23	19.17
19_2	Slovenia_2_RedTill	Green manure	Bulk soil	Reduced	10 cm	10.6	Skeletic Eutric Cambisol, loamy	6.95	Loamy	40.47	43.6	15.93
20_1	Denmark_Slovenia_KMC	Microbial consortium	NA	NA	NA	NA	Sandy soil	NA	Sandy	NA	NA	NA
