# Supplementary material for: Abundance, classification and genetic potential of Thaumarchaeota in metagenomes of European agricultural soils: a meta-analysis
Source: Environ Microbiome. 2023 Mar 30;18:26. doi: 10.1186/s40793-023-00479-9 (PMC10064710; doi:10.1186/s40793-023-00479-9)
Supplement: Supplementary file 3 — Additional file 3. Distribution of Thaumarchaeota subtaxa per soil location: Sankey diagrams of the Thaumarchaeota subtaxa distribution shown for all soil locations. [file 40793_2023_479_MOESM3_ESM.pdf]

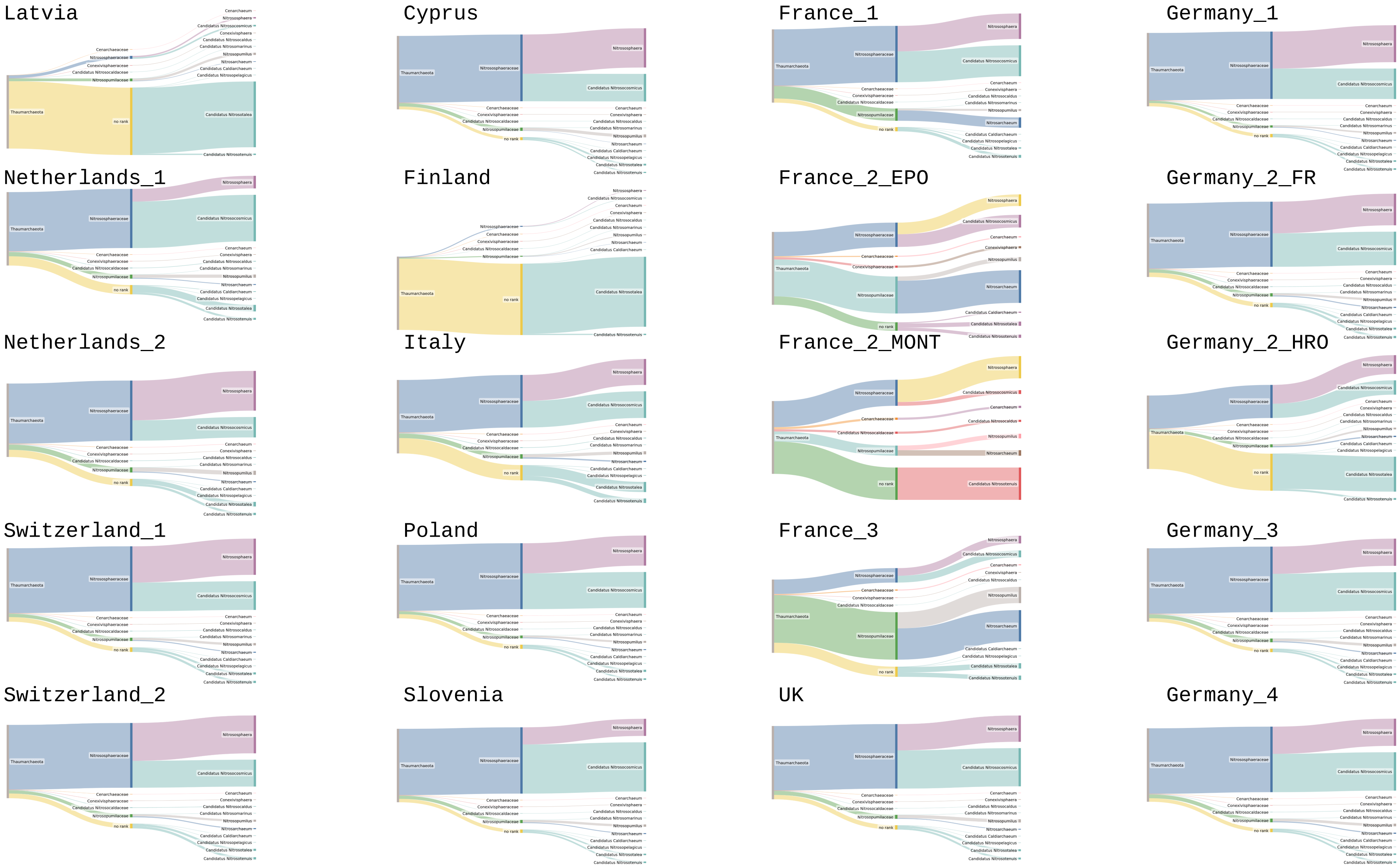

Latvia
Cyprus
France_1
Germany_1
Netherlands_1
Finland
France_2_EPO
Germany_2_FR
Netherlands_2
Italy
France_2_MONT
Germany_2_HRO
Switzerland_1
Poland
France_3
Germany_3
Switzerland_2
Slovenia
UK
Germany_4
